# Supplementary material for: Preserving Insulin Secretion in Diabetes by Inhibiting VDAC1 Overexpression and Surface Translocation in β Cells
Source: Cell Metab. 2019 Jan 8;29(1):64–77.e6. doi: 10.1016/j.cmet.2018.09.008 (PMC6331340; doi:10.1016/j.cmet.2018.09.008)
Supplement: Document S1. Figures S1–S7 and Tables S1 and S2 [file mmc1.pdf]

Cell Metabolism, Volume *29*

Supplemental Information

Preserving Insulin Secretion in Diabetes by Inhibiting VDAC1 Overexpression and Surface Translocation in β Cells

Enming Zhang, Israa Mohammed Al-Amily, Sarheed Mohammed, Cheng Luan, Olof Asplund, Meftun Ahmed, Yingying Ye, Danya Ben-Hail, Arvind Soni, Neelanjan Vishnu, Pradeep Bompada, Yang De Marinis, Leif Groop, Varda Shoshan-Barmatz, Erik Renström, Claes B. Wollheim, and Albert Salehi

Supplemental Information

Preserving insulin secretion in diabetes by inhibiting VDAC1 overexpression and surface translocation in β-cells

Enming Zhang[1], Israa Mohammed Al-Amily[1], Sarheed Mohammed[1], Cheng Luan[1], Olof Asplund[1], Meftun Ahmed[2], Yingying Ye[1], Danya Ben-Hail[3], Arvind Soni[1], Neelanjan Vishnu[1], Pradeep Bompada[1], Yang De Marinis[1], Leif Groop[1,4], Varda Shoshan-Barmatz[3], Erik Renström[1], Claes B. Wollheim[1,4,6]* and Albert Salehi [1,6,7]**

[1]Department of Clinical Science, Malmö, University of Lund, Malmö, Sweden [2]Present address, Acandemic hospital Uppsala University, Uppsala, Sweden [3]Department of Life Sciences and the National Institute for Biotechnology in the Negev, Ben-Gurion University of the Negev, Beer-Sheva 84105, Israel, [4]Finnish Institute for Molecular Medicine, Helsinki University, Helsinki, Finland, [5]Department of Cell Physiology and Metabolism, University Medical Centre, 1 rue Michel-Servet, Geneva 4, Switzerland

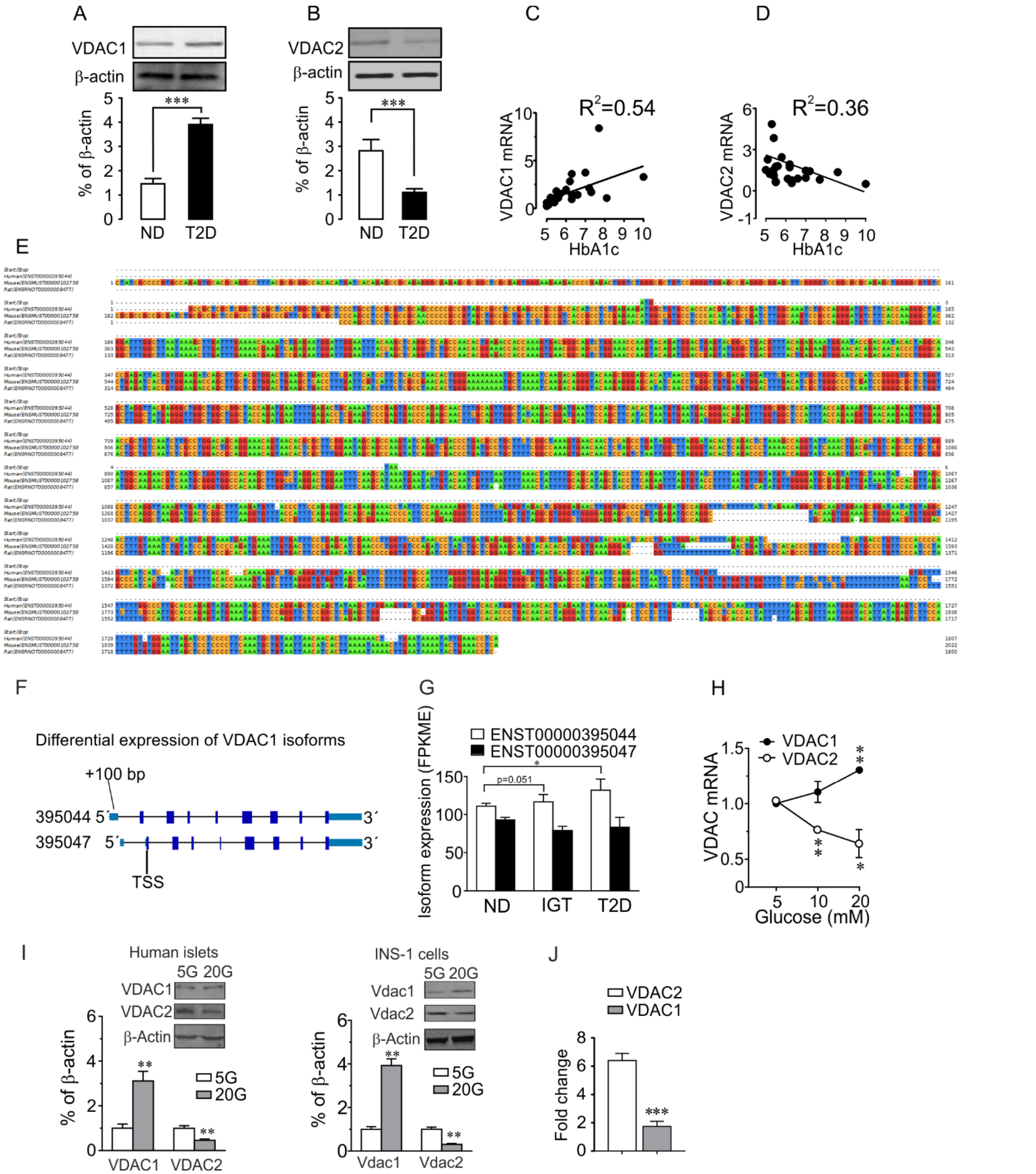

Figure S1 (related to Figure 1). A, B, Representative Western blots and fold change by densitometry normalized to β-actin (mean ± SEM) for VDAC1 (A) and VDAC2 (B) in islet extracts from non-diabetic (ND) and T2D donors (n = 8 donors in each group). C, Correlation between islet *VDAC1* mRNA and donor HbA1c in ND+T2D excluding the four metformin-treated (n=26), (*cf.* Figure 1B insert), P<0.005. D, Correlation between islet *VDAC2* mRNA and donor HbA1c in ND+T2D excluding the four metformin-treated (n=26) (*cf.* Figure 1D insert), *P<0.05. E, Alignment of the human, mouse and rat VDAC1genes. Note the difference in exon 1 in mouse on the one hand and human and the rat on the other. Alignments to the human *VDAC1* gene on chromosome 5 were visualized using the Integrative Genomics Viewer. F, Scheme of the two most abundant *VDAC1* transcript variants (ENST00000395044 and ENST00000395047) in human islets analyzed by RNA sequencing. G, Increased expression of *VDAC1* transcript variant ENST00000395047 in ND islets and those of donors with impaired glucose tolerance (IGT) and T2D as analyzed by RNA sequencing (ND=181 donors, IGT=19, T2D=16 donors). H, *VDAC1* and *VDAC2* mRNA expression in human islets cultured at 5, 10 or 20 mM glucose for 72h. Mean±SEM from two different donors with three replicates each are shown. I, Comparison of VDAC1 and VDAC2 protein levels inhuman islets and INS-1 cells after culture at 5 or 20 mM glucose for 72h. Top, representative Western blots bottom quantification of results from four different donors and from four different INS-1 cells experiments. Mean+SEM of four separate experiments normalized to the values in 5 mM glucose for islets and INS-1 cells, respectively. J, Vdac1 and Vdac2 protein detection in INS-1 cells by a rabbit polyclonal antibody, targeting a common epitope of the proteins. Mean±SEM of four separate observation. *P<0.01, ***P<0.0001.

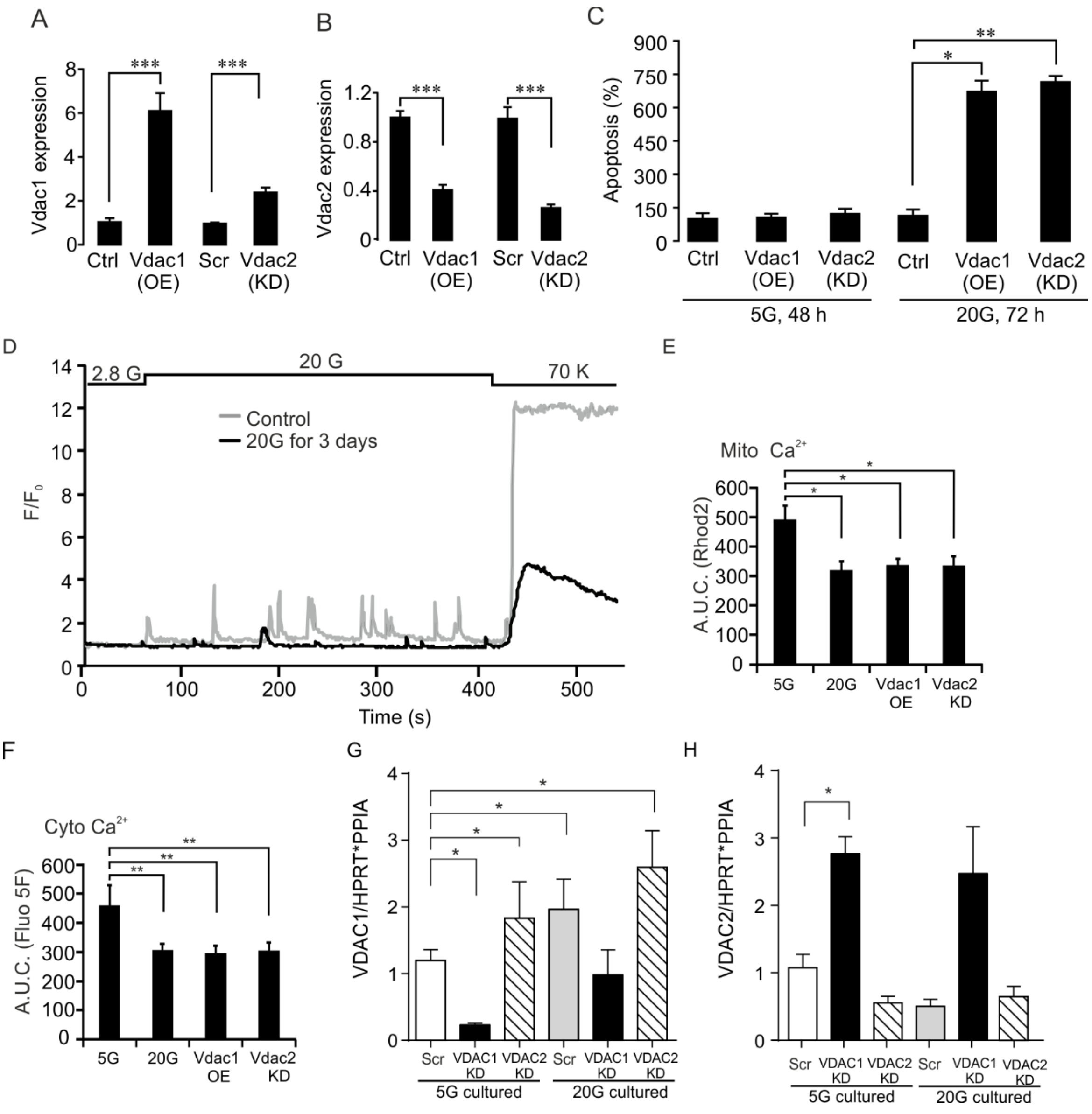

Figure S2 (related to Figure 2). A,B, Reciprocal changes in *Vdac1* (A) and *Vdac2* transcript (B) expression after overexpression of *Vdac1* (OE) and knock-down of *Vdac2* (KD) in INS-1 cells. Data are expressed as mean ± SEM (n = 9 different experiments). C, *Vdac1* overexpression (OE) or *Vdac2* knock-down (KD)-induced apoptosis measured in INS-1 cells either directly after 48 h of culture in 5 mM glucose or after an additional 72h culture in 20 mM glucose. Apoptosis is expressed as % of the control values measured in cells cultured at 5 mM glucose. Results are from three independent experiments performed with 2-3 technical replicates. D, mitochondrial (Mito) and cytosolic (Cyto) Ca^{2+} in INS-1 cells cultured at 5 or 20 mM glucose as well as after overexpression (OE) of *Vdac1* or knock-down (KD) of *Vdac2*. The cells were cultured 3 days at either 5 or 20 mM glucose. VDAC1 (OE) and VDAC2 (KD) were performed in cells cultured at 5 mM glucose. At the end of the culture period, the cells were loaded with Rhod-2 (0.75 uM) and Fluo-5F (0.5 uM) for Mito Ca^{2+} and Cyto Ca^{2+} respectively, as indicated in STAR Methods. The basal glucose concentration (2.8 mM) was raised to 20 mM glucose followed by addition of 70 mM K^+ as indicated in the figure. Representative traces of mito Ca^{2+} under control and glucotoxic conditions with indicated additions (20 mM glucose and 70 mM K^+). E, Mito Ca^{2+} as area under the curve (AUC) after acute stimulation with 20 mM glucose. F, Cyto Ca^{2+} expressed as AUC after acute stimulation with 20 mM glucose. Data are mean ± SEM of three separate experiments. Mito and Cyto Ca^{2+} were monitored simultaneously in respectively 30 cells (5 mM glucose culture), 28 cells (20 mM) culture, 27 cells with *Vdac1* OE and 29 after *Vdac2* KD. G, *VDAC1* mRNA levels in human islets after *VDAC1*-KD or *VDAC2*-KD. H, Same as in G for *VDAC2* mRNA levels. Data are mean ± SEM from three donors with 3 technical replicates in each. *P<0.05, **P<0.01, ***P<0.001.

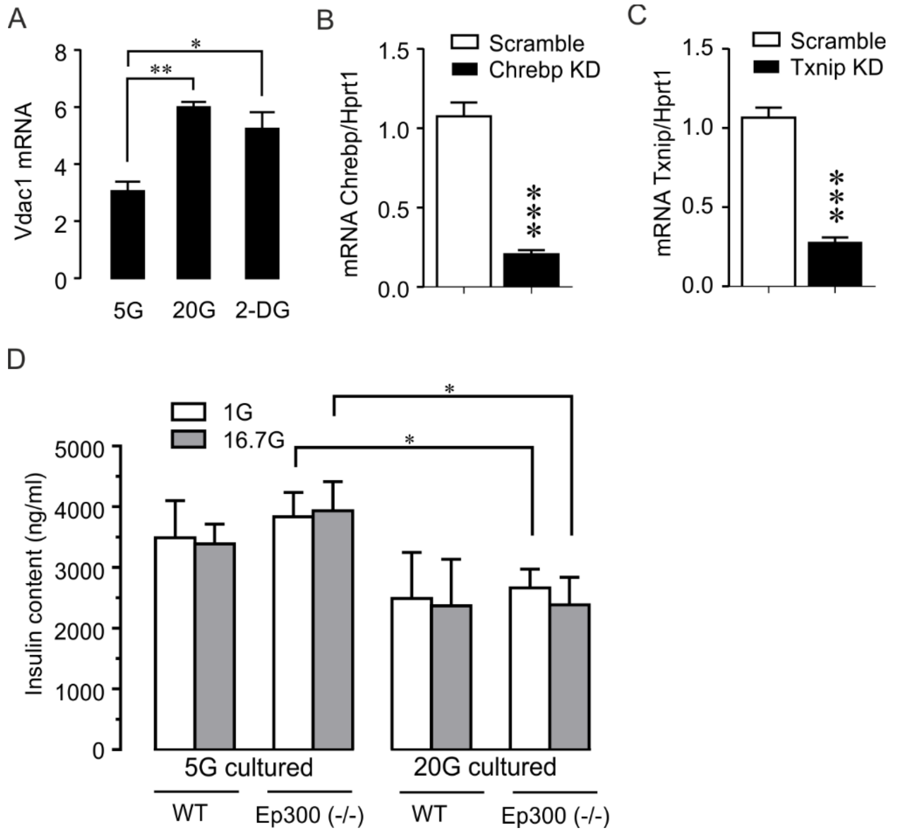

Figure S3 (related to Figure 3). A, 2-deoxy-glucose (2-DG) (15 mM) mimics the effects of glucotoxicity on *Vdac1* mRNA expression in INS-1 cells cultured at 5 mM glucose for 72h. Data are mean ± SEM from three independent experiments. B, Chrebp mRNA after knock-down in INS-1 cells (n=4 different experiments). C, Txnip mRNA after knock-down in INS-1 cells (n=4 different experiments). D, insulin content in INS-1 (Ep300(-/-) and control cells for the experiments in Figure 3G. (n= 3-4). *P<0.05, **P<0.01, ***P<0.001.

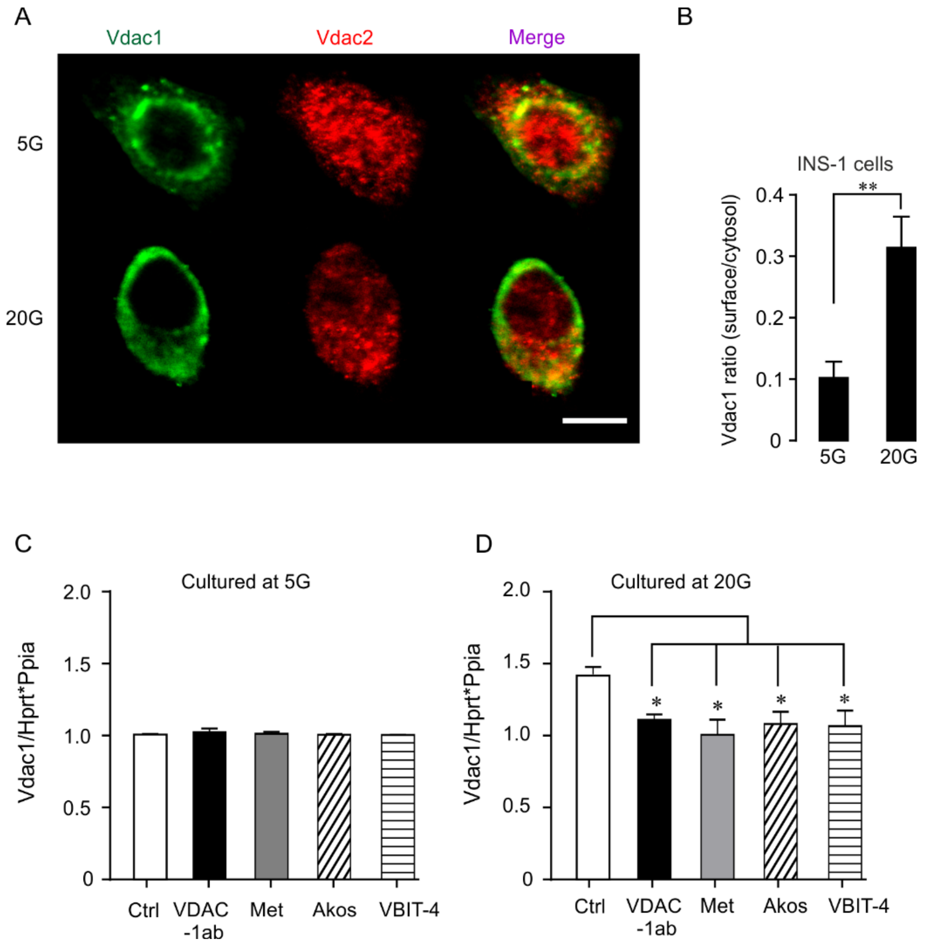

Figure S4 (related to Figure 4). A, Representative confocal images of Vdac1 or Vdac2 in INS-1 cells cultured at 5 (5G) or 20 mM glucose (20G) for 72h. The cells were double immunostained for Vdac1 and Vdac2. B, Cell surface expression of Vdac1 is calculated as the ratio of Vdac1 within 500 nm from the cell membrane over cytosolic Vdac1 (n= 7 replicates in each group from three independent experiments). C, D, VDAC1 inhibitors prevent glucotoxicity-evoked Vdac1 induction. Vdac1 expression in the presence or absence of VDAC1 antibody (VDAC1ab, 10 nM), metformin (20 µM) (Met), AKOS022075291 (20 µM) (AKOS) or VBIT-4 (20 µM) in INS-1 cells cultured for 72 h at either 5 mM (C) or 20 mM glucose (D). Results are mean ± SEM of 3 independent experiments with four technical replicates. *P<0.01, **P<0.001.

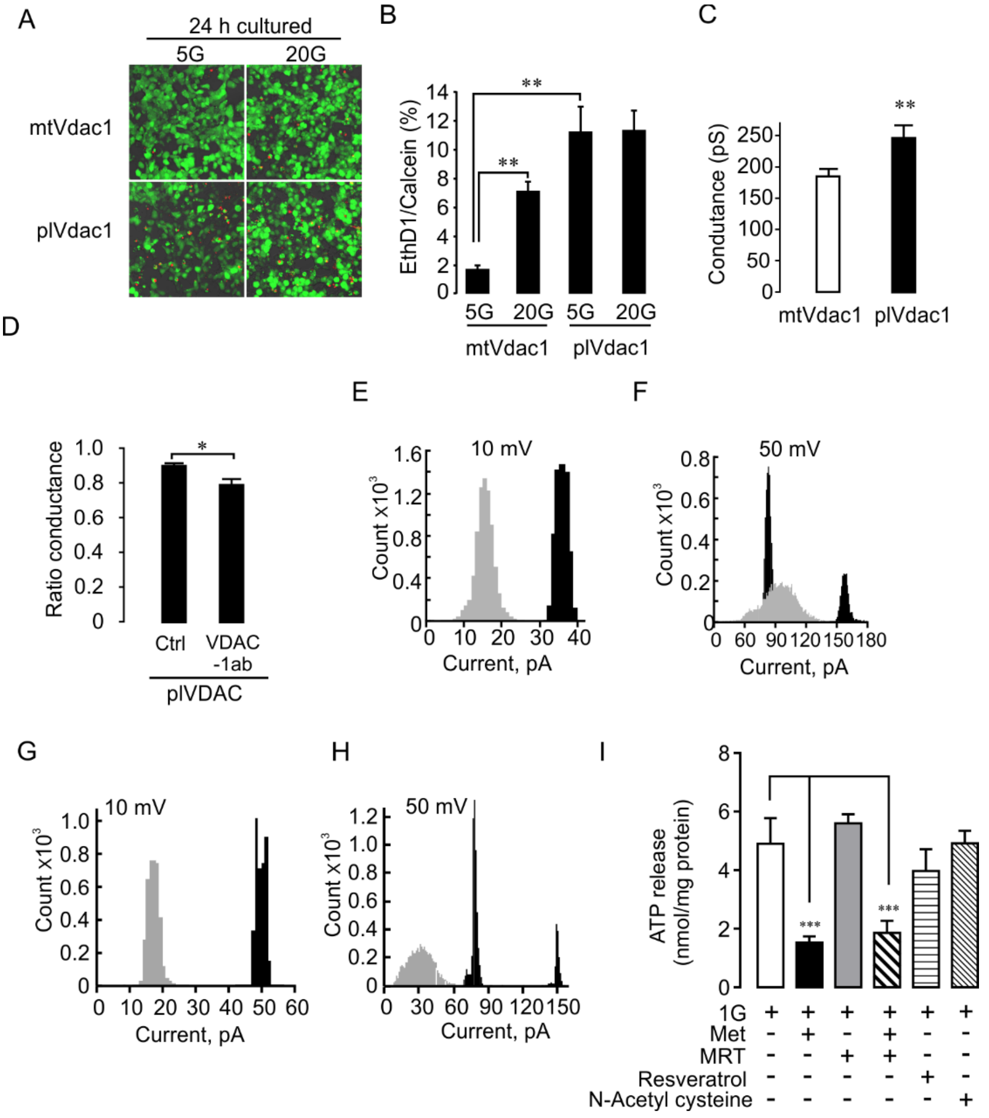

Figure S5 (related to Figure 5). A, Overexpression of plasma membrane *Vdac1* (*plVdac1*) causes cell death in INS-1 cells. Representative confocal images acquired from INS-1 cells transfected with mitochondrial *Vdac1* (*mtVdac1*) or *plVdac1* and cultured with either 5 mM (5G) or 20 mM glucose (20G) for 24h. Green (Calcein) and red (Ethidium homodimer-1, EthD1) indicate live and dead cells respectively. B, Average of ratios calculated by division of EthD1 intensity to calcein intensity under the same conditions as in A. Data are mean±SEM from three independent experiments. C, Membrane conductance recorded by the whole cell patch-clamp technique in INS-1 cells overexpressing *mtVdac1* or *plVDAC1*. Mean ± SEM of 10 cells each. D, Ratio of membrane conductance after and before acute additions of extracellular solution in INS-1 cells overexpressing *plVdac1*, without and with either VDAC1-ab or metformin. Mean ± SEM of 11 cells in each group are shown. E,F,G,H, VDAC1 conductance in planar lipid bilayer experiments. Current amplitude histograms of the experiments in Fig 5E at +10 mV (E) or 50 mV (F), before (dark grey) and 10 min after the addition of metformin (light grey) are shown. Current amplitude histograms of the experiments with VBIT-4 in Fig 5F at 10 mV (G) or, 50 mV (H), before (dark grey) and 10 min after the addition of 40 µM VBIT-4 (light grey) are shown. I, The metformin-inhibited (Met) ATP release in plVDAC1 transfected INS-1 cells is neither attenuated by an AMPK inhibitor (MRT199665, 5 µM) (MRT) (Clark et al., 2012) nor mimicked by the antioxidants resveratrol (20 µM) or N-Acetyl cystein (N-NAC 100 µM). Data are mean ± SEM of 4 independent experiments. **P<0.01.

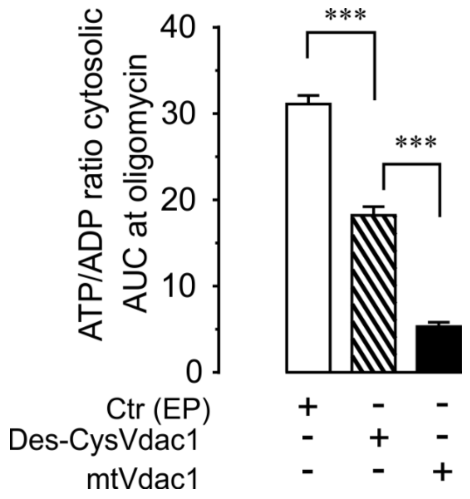

Figure S6 (related to Figure 6). The Area under curve (AUC) of mitochondrial ATP production in INS-1 cells blocked by oligomicyn (0.6 mg/ml) for the experiments shown in fig. 6E where cells express empty plasmid (EP), mtVdac1 or Des-CysVdac1. Data are mean ± SEM of 6 independent experiments with 5-10 cells in each.

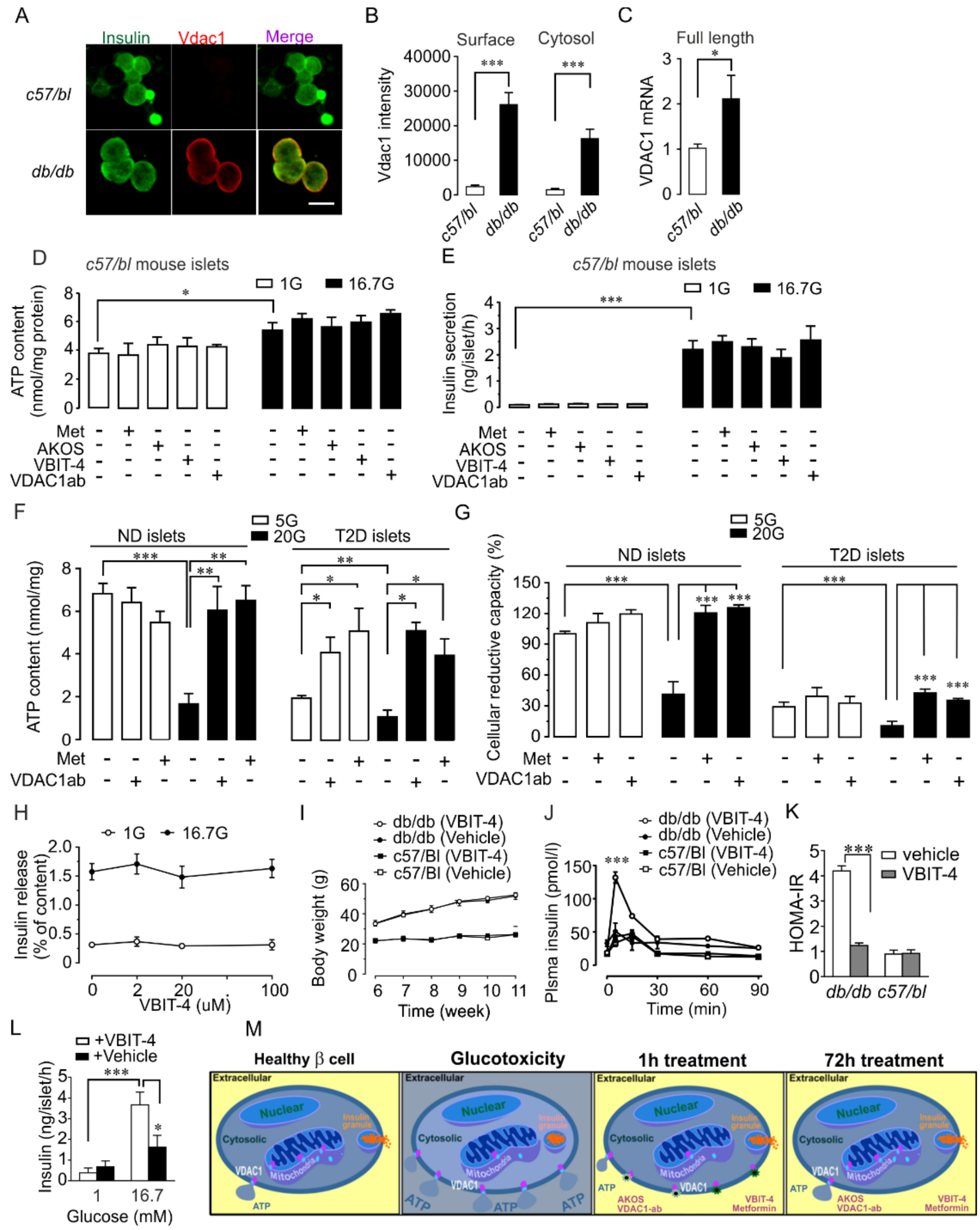

Figure S7 (related to Figure 7). Aberrant localization of VDAC1 and effects of its blockade in *db/db* mouse islets. A, Representative confocal images showed that Vdac1 is expressed predominantly on the surface of β-cells in *db/db* mouse islets (scale bar: 10 um). B, Surface and cytosolic mean intensity of Vdac1 in β-cells were measured. Results from 23 (*c57/bl*) and 25 (*db/db*) islets isolated from 8 mice of each genotype are shown. Data are mean ± SEM. C, *Vdac1* mRNA measured by qPCR in *c57/bl* and *db/db* mouse islets. D,E, VDAC1 antibody (10 nM), metformin, AKOS022075291 and VBIT-4 (all at 20 μM) do not affect ATP content (D) or GSIS (E, in *c57/bl*) islets. Islets isolated from four *c57/bl* mice were incubated separately in a single experiment. Data are mean ± SEM. F, ATP content in isolated islets from non-diabetic (ND) and T2D organ donors after culture for 72h at 5 (5G) or 20 mM glucose (20G) in the presence or absence of VDAC1 antibody (10 nM) (VDAC1ab) or metformin (Met, 20 μM). Note the improved ATP content in glucotoxic condition and T2D islets. Data are mean ± SEM of 5 non-diabetic and 3 T2D donors. G, Cellular reductive capacity (formazone production) measured in non-diabetic or T2D islets (F) after 5 or 20 mM glucose culture as in F. Mean ± SEM of 5 non-diabetic and 3 T2D donors are shown. H, Effect on insulin secretion of different concentrations of VBIT-4 added during 1 h incubation in the presence of 1 or 16.7 mM glucose (16.7G) in ND human islets. Mean+SEM of two experiments with 1 donor each. I, Body weight of *db/db* and *c57/bl* mice treated with VBIT-4 (25 mg/kg ip) or vehicle for 5 weeks. J, Plasma insulin during IPGTT (2 g/kg) of the experiments shown in Fig. 7H. Mean±SEM 12 *db/db* in each group and 5-6 c57/bl in each group is shown. K, Insulin sensitivity in *db/db* mice is improved by VBIT-4. HOMA-IR was calculated from the results shown in figures 7H and S7J. Mean+SEM of 12 *db/db* (each group) and 5-6 *c57/bl* mice are shown. L, Glucose-stimulated insulin secretion from isolated islets of *db/db* mice treated for 5 weeks with daily IP injections of VBIT-4 (25 mg/kg) or vehicle. The islets were incubated for 1h at 1 (1G) or 16.7 mM glucose (16.7G). Data are mean ± SEM of 6 mice in each group with 2 technical replicates. * $p<0.05$, ** $p<0.01$, *** $p<0.001$. M, Schematic illustration of VDAC1 plasma membrane expression in β-cells of T2D islets causing cellular ATP depletion with consequent blunting of GSIS. Inhibitors of VDAC1 (VDAC1 Antibody, VBIT-4, AKOS and metformin) block ATP depletion when added acutely and restore GSIS. Moreover, 72h presence of these inhibitors prevents the VDAC1 upregulation and mistargeting to the β-cell plasma membrane under glucotoxic conditions. This effect also provides an explanation for the protracted increase in blood glucose of *db/db* mice after cessation of VBIT-4 treatment (*cf*. Figure 7G).

Table S1 (related to Figure 1): Donors grouping information based on the HbA1c and history of T2D. Mean±sem is shown.

	Number	Gender	Age (year)	BMI (index)	Hb1Ac (%)
ND	10	Male	55.7±1.5	23.7±0.2	5.5±0.04
	5	Female	58±1.2	23.2±0.34	5.6±0.06
IGT	1	Male	68	29.3	6.1
T2D	10	Male	62.6±1.9	26.5±0.9	7.0±0.3
	5	Female	59.8±3.1	30.2±1.0	6.8±0.2
