# Supplementary material for: Preserving Insulin Secretion in Diabetes by Inhibiting VDAC1 Overexpression and Surface Translocation in β Cells
Source: Cell Metab. 2019 Jan 8;29(1):64–77.e6. doi: 10.1016/j.cmet.2018.09.008 (PMC6331340; doi:10.1016/j.cmet.2018.09.008)
Supplement: Document S2. Article plus Supplemental Information [file mmc2.pdf]

Clinical and Translational Report

Cell Metabolism

Preserving Insulin Secretion in Diabetes by Inhibiting VDAC1 Overexpression and Surface Translocation in β Cells

Graphical Abstract

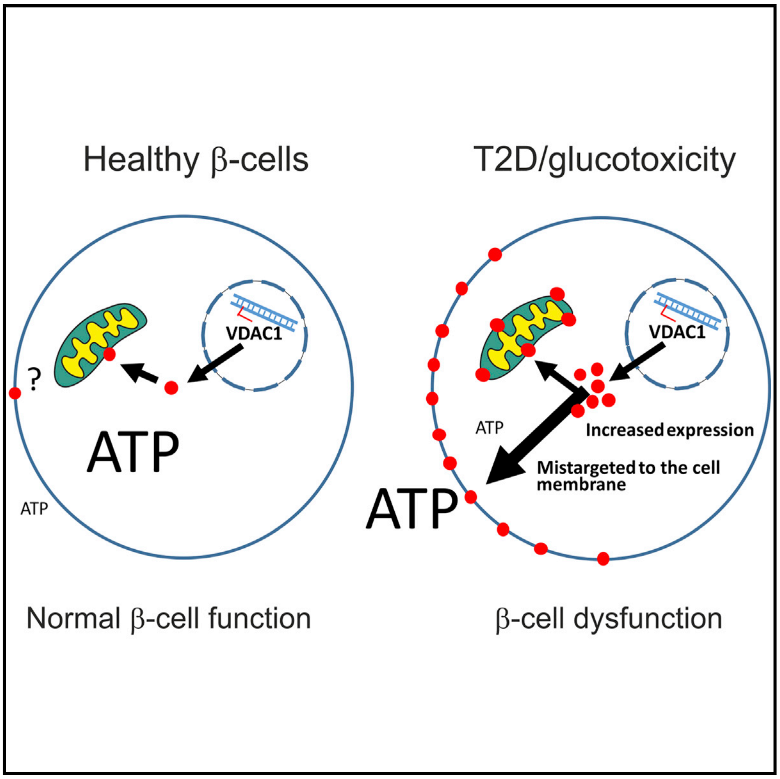

Authors

Enming Zhang, Israa Mohammed Al-Amily, Sarheed Mohammed, ..., Erik Renström, Claes B. Wollheim, Albert Salehi

Correspondence

claes.wollheim@unige.ch (C.B.W.), s_albert.salehi@med.lu.se (A.S.)

In Brief

Zhang et al. report on the role of the ATP-conducting mitochondrial outer membrane voltage-dependent anion channel-1 (VDAC1) in β cell glucotoxicity preceding diabetes. VDAC1 upregulation leads to ATP depletion and impaired insulin secretion. VDAC1 inhibitors, including metformin, restore insulin secretion in T2D islet donors and prevent hyperglycemia in diabetic mice.

Highlights

- Hyperglycemia increases VDAC1 expression and its mistargeting to the β cell surface
- VDAC1 surface expression causes ATP loss and β cell dysfunction in T2D islets
- VDAC1 inhibition restores β cell function and prevents hyperglycemia in db/db mice
- Metformin preserves β cell function by directly inhibiting VDAC1 conductance

Zhang et al., 2019, Cell Metabolism *29*, 64–77
January 8, 2019 © 2018 The Authors. Published by Elsevier Inc.
https://doi.org/10.1016/j.cmet.2018.09.008

CellPress

Cell Metabolism
Clinical and Translational Report

Preserving Insulin Secretion in Diabetes by Inhibiting VDAC1 Overexpression and Surface Translocation in β Cells

Enming Zhang,[1] Israa Mohammed Al-Amily,[1] Sarheed Mohammed,[1] Cheng Luan,[1] Olof Asplund,[1] Meftun Ahmed,[5] Yingying Ye,[1] Danya Ben-Hail,[2] Arvind Soni,[1] Neelanjan Vishnu,[1] Pradeep Bompada,[1] Yang De Marinis,[1] Leif Groop,[1,3] Varda Shoshan-Barmatz,[2] Erik Renström,[1] Claes B. Wollheim,[1,4,6,*] and Albert Salehi[1,6,7,*]
[1]Department of Clinical Sciences, Malmö, Lund University, Jan Waldenströms Gata 35, Malmö 214 28, Sweden
[2]Department of Life Sciences and the National Institute for Biotechnology in the Negev, Ben-Gurion University of the Negev, Beer-Sheva 84105, Israel
[3]Finnish Institute for Molecular Medicine, Helsinki University, Helsinki, Finland
[4]Department of Cell Physiology and Metabolism, University Medical Centre, 1 rue Michel-Servet, Geneva 4, Switzerland
[5]Academic Hospital Uppsala University, Uppsala, Sweden
[6]These authors contributed equally
[7]Lead Contact
*Correspondence: claes.wollheim@unige.ch (C.B.W.), s_albert.salehi@med.lu.se (A.S.)
https://doi.org/10.1016/j.cmet.2018.09.008

SUMMARY

Type 2 diabetes (T2D) develops after years of prediabetes during which high glucose (glucotoxicity) impairs insulin secretion. We report that the ATP-conducting mitochondrial outer membrane voltage-dependent anion channel-1 (VDAC1) is upregulated in islets from T2D and non-diabetic organ donors under glucotoxic conditions. This is caused by a glucotoxicity-induced transcriptional program, triggered during years of prediabetes with suboptimal blood glucose control. Metformin counteracts *VDAC1* induction. VDAC1 overexpression causes its mistargeting to the plasma membrane of the insulin-secreting β cells with loss of the crucial metabolic coupling factor ATP. VDAC1 antibodies and inhibitors prevent ATP loss. Through direct inhibition of VDAC1 conductance, metformin, like specific VDAC1 inhibitors and antibodies, restores the impaired generation of ATP and glucose-stimulated insulin secretion in T2D islets. Treatment of db/db mice with VDAC1 inhibitor prevents hyperglycemia, and maintains normal glucose tolerance and physiological regulation of insulin secretion. Thus, β cell function is preserved by targeting the novel diabetes executer protein VDAC1.

INTRODUCTION

Type 2 diabetes (T2D) is steadily increasing and represents a worldwide health problem. The majority of cases occur secondary to obesity with its associated insulin resistance. T2D develops after years of prediabetes, or impaired glucose tolerance (IGT). The disease becomes manifest when insulin resistance is no longer compensated by augmented insulin secretion (Aroda et al., 2017; Ligthart et al., 2016; Weir and Bonner-Weir, 2004). Accordingly, monitoring the insulin secretory capacity of pancreatic β cells best predicts future diabetes (Lyssenko et al., 2008). During prediabetes, blood glucose values gradually increase, exerting harmful effects on a wide variety of organs, including the cardiovascular system and the β cells in the pancreatic islets, so-called glucotoxicity (Wajchenberg, 2007; Weir and Bonner-Weir, 2004). Lifestyle modifications can prevent the outbreak of T2D and revert early stages of the disease but are difficult to implement. No current medication can efficiently preserve β cell function or prevent T2D (Aroda et al., 2017). Among the commonly used T2D drugs, metformin acts mainly, but not exclusively, by suppressing glucose production in the liver (reviewed in Foretz et al., 2014), while thiazolidinediones enhance peripheral insulin sensitivity and sulfonylureas force the secretion of more insulin from the already stressed β cells (Wajchenberg, 2007). Additional antidiabetic drugs have been introduced. Among these, glucagon-like peptide-1 (GLP-1) analogs and dipeptidyl peptidase-4 inhibitors enhance insulin secretion, while sodium-glucose cotransporter-2 inhibitors lower blood glucose by preventing renal glucose reabsorption (Palanisamy et al., 2018).

Dysfunction of the β cells with impaired glucose-stimulated insulin secretion (GSIS) is the main defect in T2D, since the reduction in β cell mass varies greatly between different studies (for review, see Marselli et al., 2014; Meier and Bonadonna, 2013). The β cell is particularly sensitive to caloric overload, as GSIS is attenuated after glucose infusion in healthy subjects (Boden et al., 1996) and after culture of human islets under glucotoxic conditions (Masini et al., 2014). Genetic factors predispose for β cell decompensation, and more than 100 T2D risk genes have been described, most of which encode β cell proteins (Fadista et al., 2014). Nonetheless, even combined, these risk alleles only explain the disease predisposition in 10%–15% of cases. Therefore, attention has focused on epigenetic changes conferred by high caloric intake and other diabetes risk factors (Nilsson and Ling, 2017). In particular, the stress-induced gene thioredoxin-interacting protein (*TXNIP*) is upregulated in islets

 © 2018 The Authors. Published by Elsevier Inc.
This is an open access article under the CC BY license (http://creativecommons.org/licenses/by/4.0/).

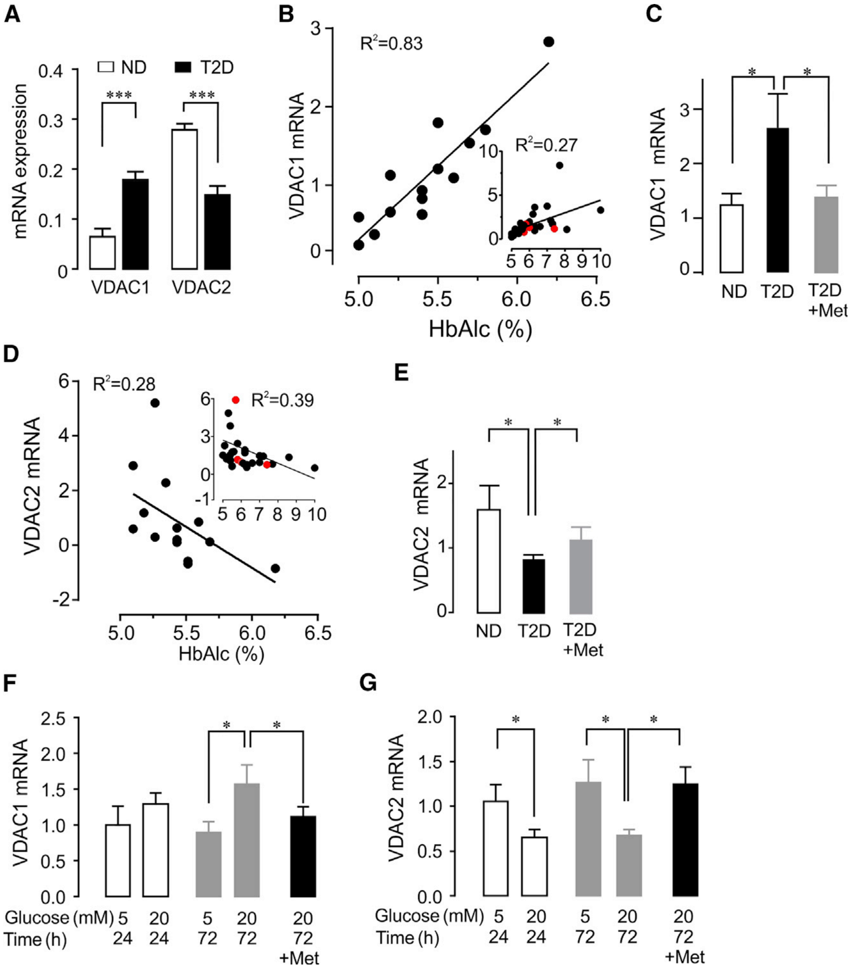

Figure 1. Expression of VDAC1 and VDAC2 in Human Pancreatic Islets

(A) *VDAC1* and *VDAC2* mRNA levels in islets from non-diabetic (ND) and T2D donors. Mean ± SEM of 19 ND and 18 T2D.

(B) Positive correlation between islet *VDAC1* mRNA and donor HbA1c in ND (HbA1c < 6.0%) (n = 15; R^2 = 0.83, p < 0.005); insert, correlation for ND + T2D, n = 30 including the four metformin-treated (red dots), R^2 = 0.27; p < 0.05.

(C) *VDAC1* expression in islets from ND (n = 15), all T2D (n = 15), and four of these T2D with documented metformin therapy.

(D) Negative correlation between islet *VDAC2* mRNA and donor HbA1c in ND (n = 14; R^2 = 0.28; p < 0.05). Correlation for ND + T2D: n = 30 including the four metformin-treated (red dots), R^2 = 0.39; p < 0.05 (insert).

(E) *VDAC2* expression in islets from ND (n = 14), all T2D (n = 15), and four of these T2D with documented metformin therapy.

(F and G) Glucotoxic condition (20 mM culture, 24 and 72 hr) mimics the T2D profile of *VDAC1* expression in human islets. Metformin (20 μM) prevents the *VDAC1* induction at 72 hr (F) and *VDAC2* suppression (G) (n = 3–5 donors).

of T2D organ donors and in β cells exposed to glucotoxic conditions (Bompada et al., 2016; Shalev, 2014). Its deletion prevents diabetes in rodents (Shalev, 2014). Glucose induces TXNIP expression by increasing the epigenetic histone acetylation of the *TXNIP* gene (Bompada et al., 2016; Cha-Molstad et al., 2009). However, the mechanism underlying the harmful effects of *TXNIP* induction in the β cell remains to be clarified.

ATP generated by glucose oxidation in β cell mitochondria couples metabolism to plasma membrane depolarization, which increases cytosolic Ca^{2+} and insulin exocytosis (Wiederkehr and Wollheim, 2012). This signaling cascade is impaired in T2D, mainly due to defective mitochondrial metabolism (Anello et al., 2005; Doliba et al., 2012; MacDonald et al., 2009). The voltage-dependent anion channel (VDAC) is the most abundant protein of the outer mitochondrial membrane. VDAC1 and VDAC2 determine cell life and death by regulating flux of metabolites, nucleotides, including ADP and ATP, as well as ions between the mitochondria and the cytosol, while the VDAC3 isoform is less well characterized (Naghdi and Hajnoczky, 2016; Shoshan-Barmatz et al., 2010). There is a striking comorbidity between T2D and Alzheimer's disease (AD) (Ribe and Lovestone, 2016). In AD, *VDAC1* is induced early in the disease, associated with its overexpression in the neurolemma (Fernandez-Echevarria et al., 2014). Moreover, VDAC1 antibodies protect cells from amyloid β (Aβ) peptide-induced neurotoxicity (Akanda et al., 2008; Smilansky et al., 2015). Such effects have not been reported in T2D. Therefore, we investigated the involvement of VDAC in β cell glucotoxicity. In particular, we studied the transcriptional program induced by glucose in insulinoma cells and human pancreatic islets. The role of VDAC1 in the development of hyperglycemia was also examined in the *db/db* mouse, a commonly used diabetes model. We report that VDAC1 overexpression and mistargeting to the β cell plasma membrane in T2D causes ATP loss. Direct inhibition of VDAC1 in human T2D β cells restores GSIS and prevents development of diabetes in *db/db* mice. Metformin also acutely improves GSIS by directly blocking VDAC1 channel function, a hitherto not appreciated mode of action of the antidiabetic drug.

RESULTS AND DISCUSSION

Altered VDAC Expression in T2D Islets and after Glucotoxicity

Islets from T2D organ donors (Table S1 for donor characteristics) display upregulated *VDAC1* mRNA, while *VDAC2* mRNA is repressed, compared with islets from non-diabetic (ND) donors (Figure 1A). These results were substantiated at the protein level (Figures S1A and S1B). *VDAC1* mRNA is strikingly correlated with average blood glucose during the months preceding the demise (glycated A1c, HbA1c) in ND islets (Figure 1B). When the results obtained in T2D donors are included, the correlation, albeit significant, is less marked (Figure 1B, insert).

Metformin is the most frequently used antidiabetic medication (Foretz et al., 2014). We could document four donors with

metformin therapy. The correlation between HbA1c and *VDAC1* expression was more significant when the metformin-treated donors were excluded (Figure S1C). Accordingly, the islets from the metformin-treated donors did not display increased *VDAC1* mRNA (Figure 1C). Conversely, there was a negative correlation between HbA1c and islet *VDAC2* mRNA (Figure 1D), which was only marginally changed by removal of the metformin-treated donors (Figures 1D insert, S1D, and 1E for islet mRNA levels). We identified two abundantly expressed *VDAC1* transcript variants (Figures S1E–S1G). Of note, one of these VDAC1 transcript variants appears to be induced by hyperglycemia, as the elevated expression in T2D donors is already seen in part in islets from IGT donors (Figure S1G). This suggests that impaired blood glucose control drives *VDAC1* overexpression, while *VDAC2* downregulation, albeit significant, is less pronounced. To evaluate if the changes observed in T2D islets can be linked to glucotoxicity, we cultured human islets under glucotoxic conditions (20 mM glucose for 72 hr), known to attenuate GSIS in human islets (Masini et al., 2014). Exposure to high glucose reproduces the T2D profile, increasing *VDAC1* and decreasing *VDAC2* mRNA (Figures 1F and 1G). However, culture of the islets at 10 mM glucose does not induce VDAC1, whereas *VDAC2* mRNA is suppressed (Figure S1F). It may be speculated that increased *VDAC1* requires longer time culture at high physiological glucose concentrations; while *VDAC2* suppression is more sensitive to elevated glucose. The changes in VDAC1 and VDAC2 under glucotoxic conditions were substantiated at the protein level for both human islets and rat insulinoma INS-1 cells (Figure S1I). To study the effect of metformin, we cultured ND human islets under glucotoxic conditions in the presence of 20 μM metformin, corresponding to plasma concentrations during drug treatment (Foretz et al., 2014). As shown in Figures 1F and 1G, the inclusion of metformin in the culture medium prevents the alterations in *VDAC1* and *VDAC2* mRNA levels. These results confirm the observations in metformin-treated islet donors.

Altered VDAC Expression Does Not per se Cause Apoptosis

In most investigated tissues and cells, *VDAC1* is much more abundant than *VDAC2* (Naghdi and Hajnoczky, 2016; Shoshan-Barmatz et al., 2010), while *VDAC2* mRNA is higher than VDAC1 mRNA in human β cells (Blodgett et al., 2015). Moreover, VDAC2 protein is more abundant than VDAC1 in INS-1 cells (Figure S1J). This may suggest an important role for VDAC2 in β cells. It is noteworthy that manipulations of VDAC1 and VDAC2 expression in β cells led to reciprocal changes, overexpression of *VDAC1* suppresses *VDAC2*, and silencing of *VDAC2* increases *VDAC1* (Figures S2A and S2B for INS-1 cells; Figures S2G and S2H for human islets). Therefore, altered expression of either isoform could impact on β cell function.

Metabolic and oxidative stress are known to upregulate VDAC1 and cause its oligomerization with ensuing apoptosis (Shoshan-Barmatz et al., 2017). To address this question, we measured apoptosis in INS-1 cells after overexpression of *Vdac1* or knockdown of *Vdac2*, reaching changes in VDAC protein levels similar to those of T2D islets and islets or INS-1 cells cultured under glucotoxic conditions (cf. Figure S1G). Evaluation of cell death by determining cytoplasmic nucleosomes in INS-1 cells after overexpression of *Vdac1* or *Vdac2* downregulation at 5 mM glucose shows no significant increase in apoptosis. In contrast, altered VDAC1 expression combined with 20 mM glucose culture, results in marked cell death (Figure S2C). Thus, the diabetes-associated alteration in VDAC expression profile does not per se cause β cell death, suggesting that additional insults trigger apoptosis. In this context, it should be emphasized that while loss of functional β cell mass in T2D is well documented, the contribution of β cell death is debated (Marselli et al., 2014; reviewed in Meier and Bonadonna, 2013). Therefore, we focused on the role of the two VDAC isoforms in β cell function.

Changes in VDAC Expression Impair Mitochondrial Function

We first examined insulin secretion in INS-1 cells. GSIS is markedly inhibited after *Vdac1* overexpression or *Vdac2* knockdown (Figures 2A and 2B). In T2D islets, mitochondrial dysfunction underlies the blunted GSIS (Anello et al., 2005; Doliba et al., 2012). We therefore assessed mitochondrial oxygen consumption rate (OCR). OCR is inhibited both at non-stimulatory (2.8 mM) and stimulatory (16.7 mM) glucose concentrations in cells with altered *Vdac* isoform expression (Figures 2C and 2D; see S2A and S2B for expression levels). The same phenotype with reduced OCR is reproduced by cell culture under glucotoxic conditions (Figures 2E and 2F), further implicating altered VDAC function in the β cell defect.

Glucose oxidation in the β cell mitochondria increases the crucial intracellular signaling molecules ATP and Ca^{2+}, which couple metabolism to insulin exocytosis (Doliba et al., 2012; Wiederkehr et al., 2011; Wiederkehr and Wollheim, 2012). Therefore, we next monitored glucose-induced increases in cytosolic and mitochondrial Ca^{2+} during acute stimulation. As expected from the attenuated GSIS, the increases in both cytosolic and mitochondrial Ca^{2+} evoked by glucose are markedly blunted in cells with upregulated VDAC1 or downregulated VDAC2, as well as after glucotoxic treatment (Figures S2D–S2F). Thus, these results strongly support the involvement of altered VDAC expression in the impaired metabolism-secretion coupling evoked by glucotoxicity. VDAC1 is essential in the function of endoplasmic reticulum-mitochondria contact sites (De Pinto et al., 2010; Shoshan-Barmatz et al., 2017). Decreased VDAC1 protein in these contact sites was observed in human T2D β cells (Thivolet et al., 2017). Thus, not only altered *VDAC* gene expression but also protein localization could contribute to the mitochondrial dysfunction.

Changes in VDAC Expression Explain Blunted GSIS in Human Islets

Subsequently, we investigated whether human islets can be protected from glucotoxicity by silencing VDAC1. Knockdown of *VDAC1* results in upregulation of *VDAC2* mRNA, while *VDAC2* silencing increases *VDAC1* expression as in INS-1 cells (Figures S2G and S2H). Remarkably, *VDAC1* knockdown not only prevents the suppression of *VDAC2* induced by culture in 20 mM glucose, but *VDAC2* mRNA is considerably higher than in islets maintained at 5 mM glucose (Figures S2G and S2H). To evaluate the impact of altered VDAC expression, we first measured overall cellular reductive capacity, which is conveniently monitored by formazone production from tetrazolium, reflecting β cell mitochondrial metabolism (Janjic and Wollheim, 1992). Reductive

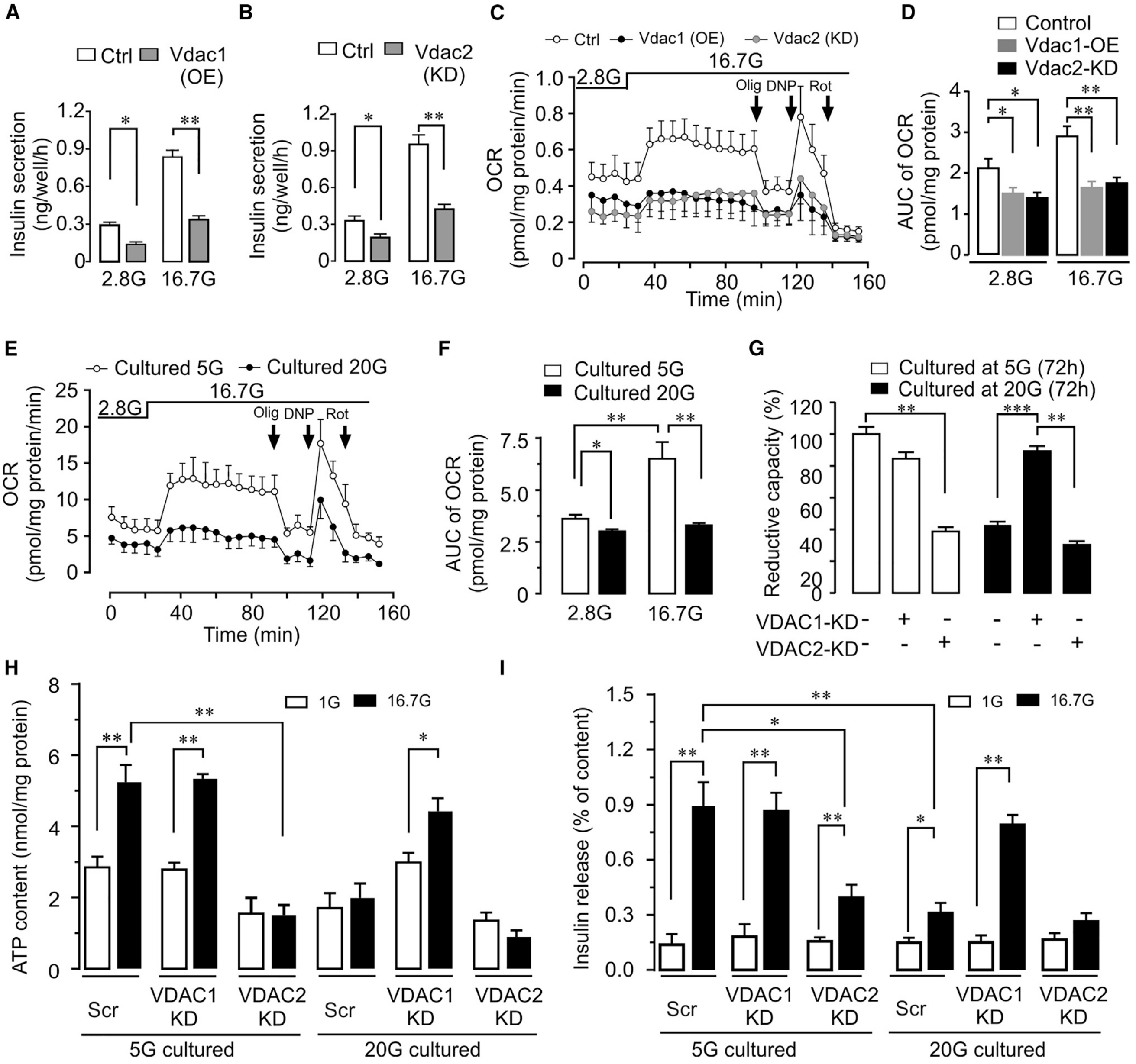

Figure 2. Impact of VDAC1 on β Cell Function

(A and B) Glucose-stimulated insulin secretion (GSIS) in INS-1 cells after overexpression of Vdac1 (OE) (A) or knockdown (KD) of Vdac2 (B) (n = 5).

(C) Oxygen consumption rate (OCR) in INS-1 cells after overexpression of Vdac1 or KD of Vdac2. Subsequent additions were as follows: oligomycin ([Olig], an inhibitor of ATP synthase) (0.4 μM), dinitrophenol ([DNP], an uncoupler) (0.4 μM), and rotenone ([Rot], an inhibitor of complex (I) (0.1 μM).

(D) Area under the curve (AUC) for the experiments in (C) (n = 5).

(E and F) OCR of INS-1 cells cultured at 5 or 20 mM glucose (72 hr) (n = 5).

(G) *VDAC1* silencing protects human islet cells from glucotoxicity-induced decrease in cellular reductive capacity (formazone production), while *VDAC2* KD is harmful. Islets from five donors (used in separate experiments) were cultured at either 5 or 20 mM glucose for 72 hr.

(H) Effect of *VDAC1* or *VDAC2* KD on ATP content of islets cultured at 5 or 20 mM glucose (72 hr) and incubated at 1 or 16.7 mM glucose for 1 hr (n = 3 donors).

(I) Insulin secretion for the same islets as in (H).

capacity in 5 mM glucose-cultured islets is not altered by *VDAC1* knockdown, which, however, completely prevents the marked impairment under the glucotoxic condition. In contrast, *VDAC2* silencing evokes similar low overall cellular reductive capacity as 20 mM glucose culture (Figure 2G). These results are substantiated by measurements of ATP content and insulin secretion after 1 hr incubation of islets after prior culture at either 5 or 20 mM glucose. In the glucotoxic islets, 16.7 mM glucose-induced increases in ATP and insulin secretion are abrogated. Like reductive capacity, silencing of *VDAC1* preserves basal

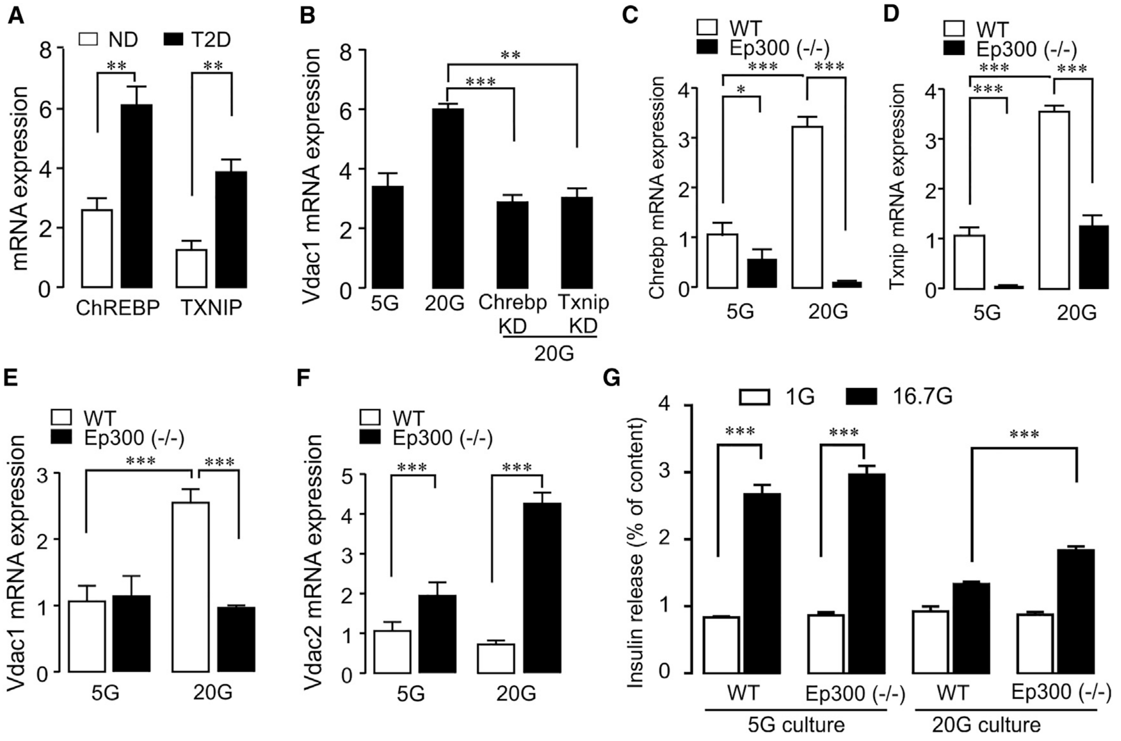

Figure 3. Transcriptional Program Causing VDAC1 Overexpression after Glucotoxicity

(A) *ChREBP* and *TXNIP* mRNA is increased in T2D islets (n = 4–5 donors).

(B) Vdac1 overexpression is blunted after silencing of either Chrebp or Txnip in 20 mM glucose-cultured INS-1 cells (n = 5).

(C) Glucose-evoked ChREBP induction is prevented in histone acetyltransferase p300 knockout INS-1 (*Ep300*$^{-/-}$) cells.

(D) Txnip expression is suppressed in INS-1 (*Ep300*$^{-/-}$) cells after 5 mM glucose culture. Txnip induction is blunted at 20 mM glucose.

(E) VDAC1 is not induced by glucotoxicity in INS-1 (*Ep300*$^{-/-}$) cells.

(F) VDAC2 is upregulated in INS-1 (*Ep300*$^{-/-}$) cells at 5 mM and further increased after culture at 20 mM glucose (n = 3–4).

(G) INS-1 (*Ep300*$^{-/-}$) cells are partially protected from blunting of GSIS after culture at 20 mM glucose (n = 3–4). Independent experiments given as n.

ATP levels and rescues glucose-evoked increases in ATP and insulin secretion. In contrast, *VDAC2* suppression reduces basal islet ATP and blunts both ATP elevation and stimulated insulin secretion (Figures 2H and 2I). Taken together, the altered VDAC expression profile in glucotoxic cells impairs mitochondrial metabolism and, as a consequence, GSIS, while the impact on β cell viability is less pronounced.

VDAC1 Induction by Epigenetic Activation of TXNIP

Next, we investigated the transcriptional program induced by glucotoxicity. It is well documented that glucotoxicity in the β cell, like oxidative stress, upregulates *TXNIP* by the induction and nuclear transfer of the carbohydrate response element-binding protein (*ChREBP*) (Poungvarin et al., 2012; Shaked et al., 2011; Shalev, 2014). This genetic program is also activated by the non-metabolizable glucose analog 2-deoxyglucose, which promotes metabolism-independent nuclear transfer of ChREBP (Shaked et al., 2011; Shalev, 2014). T2D islets display increased transcripts of both ChREBP and TXNIP (Figure 3A), confirming published results (Bompada et al., 2016; Poungvarin et al., 2012; Shalev, 2014). *Vdac1* induction by 2-deoxyglucose in INS-1 cells (Figure S3A) substantiates the involvement of ChREBP and TXNIP in *VDAC1* gene regulation by glucotoxicity. Moreover, knockdown of either *Chrebp* or *Txnip* in INS-1 cells prevents glucotoxicity-induced *Vdac1* upregulation (Figure 3B; for silencing efficacy, see Figures S3B and S3C). The transcriptional activation of *TXNIP* by glucose requires not only ChREBP dimerization with MLX, but also the recruitment of the histone acetyltransferase p300 (Ep300) for efficient transcription (Bompada et al., 2016; Cha-Molstad et al., 2009). Ep300 activity is necessary for glucose-induced *TXNIP* upregulation in human islets and INS-1 cells by altering histone acetylation marks in the *TIXNIP* gene (Bompada et al., 2016). To further characterize the roles of ChREBP and TXNIP in VDAC expression, we used INS-1 cells with Ep300 knockout (KO) established by CRISPR/Cas9 (Bompada et al., 2016). As expected, INS-1 (*Ep300*$^{-/-}$) cells exhibit extremely low *Txnip* mRNA. The repressed *Txnip* evoked significantly reduced *Chrebp* mRNA after culture at 5 mM glucose compared with wild-type (WT) INS-1 cells (Figures 3C and 3D). After 72 hr exposure to 20 mM glucose, the *Chrebp* transcript is further decreased, while *Txnip* levels are upregulated, reaching those of the 5-mM-cultured WT-INS-1 cells. Such an attenuated induction of *Txnip* was already described for the Ep300 KO cells (Bompada et al., 2016). INS-1 (*Ep300*$^{-/-}$) cells are completely protected from the glucotoxicity-induced increase in *VDAC1* mRNA (Figure 3E). Conversely, *VDAC2* mRNA in INS-1 (*Ep300*$^{-/-}$) cells is already elevated under physiological glucose and, rather than decreasing, further

increases in the high glucose condition (Figure 3F). Based on the foregoing results, low VDAC1 combined with high VDAC2 expression should preserve GSIS. Hence, INS-1 (*Ep300*$^{-/-}$) cells display similar GSIS after 5 mM glucose culture as the WT cells. The Ep300-KO cells are substantially, but not completely, protected from glucotoxicity-mediated abrogation of GSIS (Figure 3G). The incomplete restoration is not due to changes in cellular insulin content (Figure S3D). Epigenetic mechanisms have been implemented in the development of β cell dysfunction in T2D through environmental stress (Nilsson and Ling, 2017). Our results support this link, as glucotoxicity-mediated VDAC1 overexpression requires the histone acetyltransferase Ep300 for the transcriptional activation of TXNIP.

VDAC1 Overexpression Leads to Its Mistargeting to the β Cell Membrane

The impaired metabolism-secretion coupling prompted us to examine the cellular localization of VDAC1 and VDAC2. Extramitochondrial plasma membrane VDAC1 (De Pinto et al., 2010; Stadtmuller et al., 1999; Thinnes, 2015) participates in volume regulation, ATP and metabolite transport, and intrinsic mitochondrial apoptosis (Akanda et al., 2008; Okada et al., 2004; Shoshan-Barmatz et al., 2010). Remarkably, confocal microscopy reveals that VDAC1, but not VDAC2, surface expression occurs in β cells from T2D donors (Figures 4A and 4B). In ND and in the single donor of four with documented metformin therapy we could examine (*cf.* Figure 1C), VDAC1 remains intracellular (Figures 4A and 4B). VDAC1 surface expression correlates positively with the islet donor HbA1c when ND and T2D islets were pooled (Figure 4C). The surface localization may thus be a consequence of the organ donors' high plasma glucose concentrations. To further substantiate the surface localization of VDAC1, we performed triple immunofluorescence staining in pancreas sections of ND and T2D donors. VDAC1 is clearly overexpressed at the T2D β cell surface, as shown by the co-localization with the plasma membrane-associated SNARE protein SNAP-25 (Figures 4D and 4E). Similar to the observations in pancreas sections, β cells in islets isolated from a single T2D donor also show intense VDAC1 overexpression (Figure 4F). Similar results were obtained in β cell of ND islets kept in culture for 72 hr at 20 mM glucose relative to control conditions (Figure 4G) or INS-1 cells (Figures S4A and S4B). Thus, VDAC1 mistargeting to the plasma membrane may cause the impaired GSIS in glucotoxicity and T2D. It is of interest, in this context, that mitochondrial VDAC1 protein is reduced in T2D β cells (Thivolet et al., 2017). Of note, in AD, VDAC1 is also upregulated in neurons of affected brain areas early in the condition, and associated with translocation of the protein to the neurolemma (Fernandez-Echevarria et al., 2014).

VDAC1 Surface Expression Causes VDAC1 Upregulation

Subsequently, we studied the functional consequences of aberrant VDAC1 subcellular localization. The mouse *Vdac1* gene is alternatively transcribed, yielding an exon1 splice variant encoding a plasma membrane-targeted protein (plVDAC1) (Buettner et al., 2000). Such splicing is not occurring in the human and rat *VDAC1 genes* (see Figures S1E and S1F; see also Raghavan et al., 2012). We postulate that VDAC1 overexpression leads to its targeting to extra-mitochondrial locations. Plasma membrane resident VDAC1 has been documented in various mouse and human tissues with the mitochondrial surface residues facing the extracellular space (Akanda et al., 2008; Buettner et al., 2000; De Pinto et al., 2010; Okada et al., 2004; Stadtmuller et al., 1999; Thinnes, 2015). In neurons, plVDAC1 activation initiated apoptosis, prevented by antibodies directed against the extracellular N terminus of VDAC1 (Akanda et al., 2008; Smilansky et al., 2015). Therefore, we investigated whether VDAC1 antibody, VBIT-4 and AKOS022075291 (AKOS), two specific small-molecule VDAC inhibitors (Ben-Hail et al., 2016), affect glucotoxicity-evoked VDAC1 induction in INS-1 cells, like metformin in human islets (Figure 1F). Indeed, not only metformin but also all three VDAC inhibitors prevent glucotoxicity-mediated overexpression of VDAC1 mRNA (Figures S4C and S4D). Moreover, inclusion of VBIT-4 in the 20 mM glucose culture of ND islets also decreases VDAC1 protein expression (Figure 4G). Thus, VDAC1 overexpression is prevented by metformin and inhibitors of VDAC1, suggesting that, as in neurons (Akanda et al., 2008), plVDAC1 conductance is harmful and notably affecting gene transcription in β cells. Of note, in the limited number of T2D donors with documented metformin therapy, there was no VDAC1 overexpression (cf. Figure 1C). The putative protective action of metformin was substantiated by our *in vitro* experiments. Metformin at a low therapeutic concentration prevented the high glucose-evoked changes in VDAC1 and VDAC2 expression in islets and INS-1 cells. Metformin inhibits glucotoxicity-evoked TXNIP induction in both insulin-secreting (Shaked et al., 2011) and endothelial cells (Li et al., 2015), which are important targets for insulin action. In diabetic mice, VDAC1 upregulation causes apoptosis in heart coronary endothelial cells (Sasaki et al., 2012), whose malfunction is conducive in T2D cardiovascular complications. Moreover, *VDAC1* is upregulated in a model of diabetic cardiomyopathy and in cardiomyocytes cultured under glucotoxic conditions (Li et al., 2016). The action of metformin was reported to be due to stimulation of AMPK (Foretz et al., 2014), which prevents ChREBP nuclear translocation and TXNIP transcriptional activation (Li et al., 2015; Shaked et al., 2011; Shalev, 2014).

VDAC1 Mistargeting to the Cell Membrane Causes Cellular ATP Depletion

To further study the consequence of VDAC1 overexpression, we monitored ATP levels during 1-hr experiments in plVdac1-expressing INS-1 cells. Overexpression of WT Vdac1 (mt Vdac1) leads to a 3-fold increase in ATP release from the cells (Figure 5A), suggesting mistargeting of the ATP-conducting Vdac1 to the plasma membrane. This is substantiated by plVdac1 expression, which causes a 10-fold ATP loss (Figure 5A). The robust GSIS in cells transfected with control plasmid is markedly reduced in mt*Vdac1*-transfected cells and completely abolished in *plVdac1*-expressing cells (Figure 5B). Moreover, glucose (20 mM) aggravates the marginal cell death in mtVdac1 cells, while plVdac1 alone is more harmful (Figures S5A and S5B). In plVdac1 cells, the loss of ATP is rapidly (1 hr) inhibited not only by VDAC1 antibody, the VDAC inhibitors AKOS and VBIT-4, but also by metformin (Figure 5C). VDAC1 overexpression thus leads to ATP loss as a consequence of its translocation to the plasma membrane.

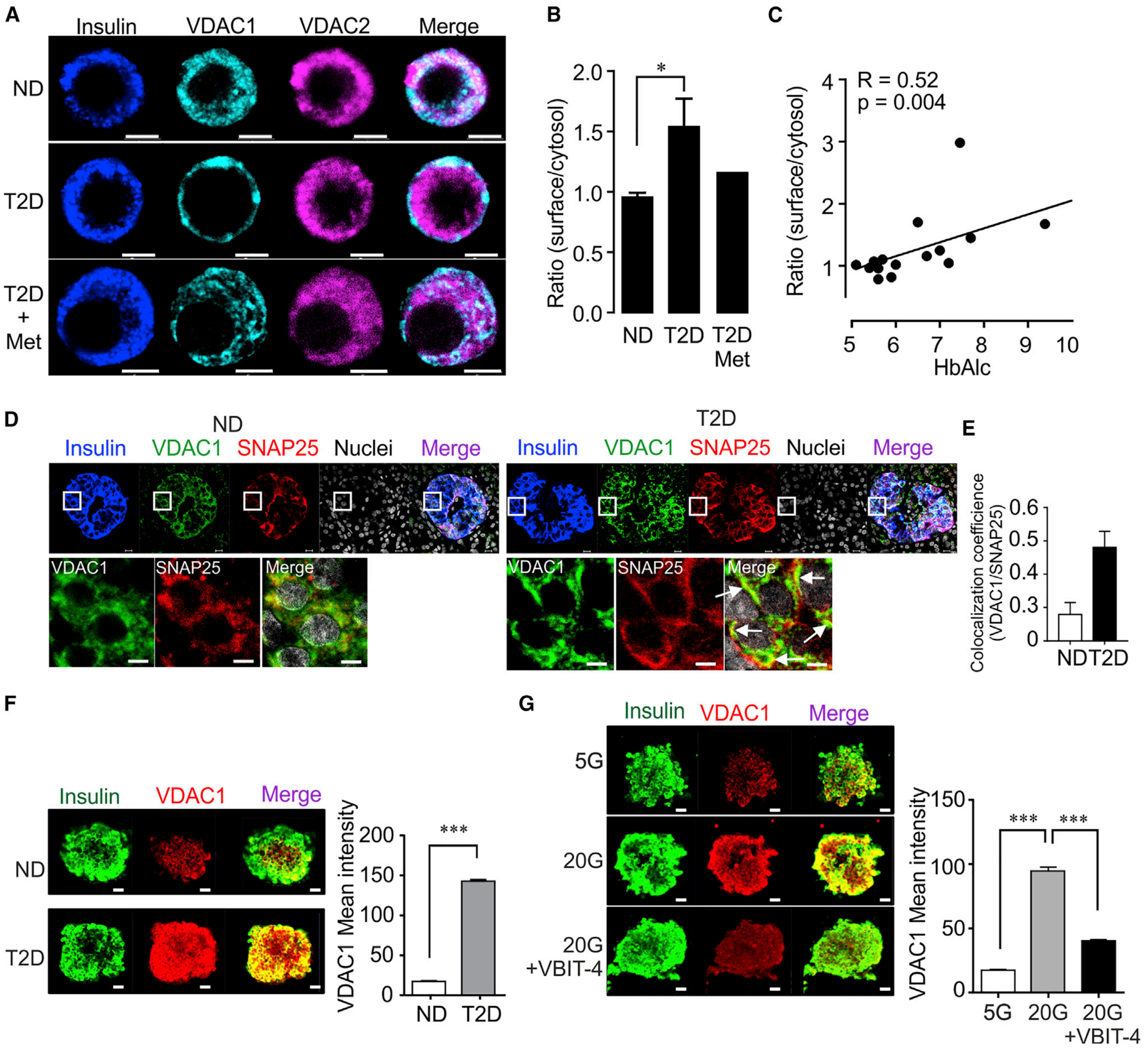

Figure 4. VDAC1 Localization in β Cells from ND and T2D Islet Donors

(A) Representative immunofluorescence images of VDAC1 and VDAC2 in human islet β cells from non-diabetic (ND) and T2D donors, one of whom had received metformin therapy. Note VDAC1 expressed prominently on the β cell surface in T2D islets.

(B) β cell surface expression of VDAC1 given as ratio of surface/cytosolic VDAC1 immunofluorescence intensity in β cells of ND or T2D (12–15 cells/donor were acquired for the analysis, 8 donors each in the ND and T2D group) and the one with metformin therapy (12 cells).

(C) Correlation between VDAC1 β cell surface expression and HbA1c values in 15 islet donors (ND and T2D).

(D) Confocal image of VDAC1 co-localization with SNAP-25 by double immunostaining in insulin-positive cells in pancreatic sections from ND and T2D donors (3 donors with 27 islets in each group). Scale bar indicates 5 μm. Arrows show co-localization of VDAC1 and SNAP-25 also magnified in the squares. Mean SNAP-25 intensity/islet was 10.2 ± 2.4 for ND and 10.5 ± 3.9 for T2D, respectively.

(E) Calculation of coefficients (VDAC1/SNAP-25) was performed using a confocal image analyzer (ZEN2012).

(F) Representative immunofluorescence images of VDAC1 expression in isolated islets from two ND and one T2D donor. Mean ± SEM of 12–20 islets from each condition were analyzed.

(G) Representative immunofluorescence images of VDAC1 expression and its co-staining with insulin-positive cells in islets from two ND donors cultured at 5 or 20 mM glucose ± VBIT-4 (20 μM) for 72 hr. Scale bar indicates 10 μm.

Metformin Inhibits VDAC1 Solute Conductance

Altered membrane permeability is further substantiated by patch-clamp experiments (Buda et al., 2013). Indeed, plVdac1-expressing INS-1 cells show 30% higher membrane conductance than mtVdac1-transfected cells (Figure S5C). The increased conductance caused by plVdac1 relative to control

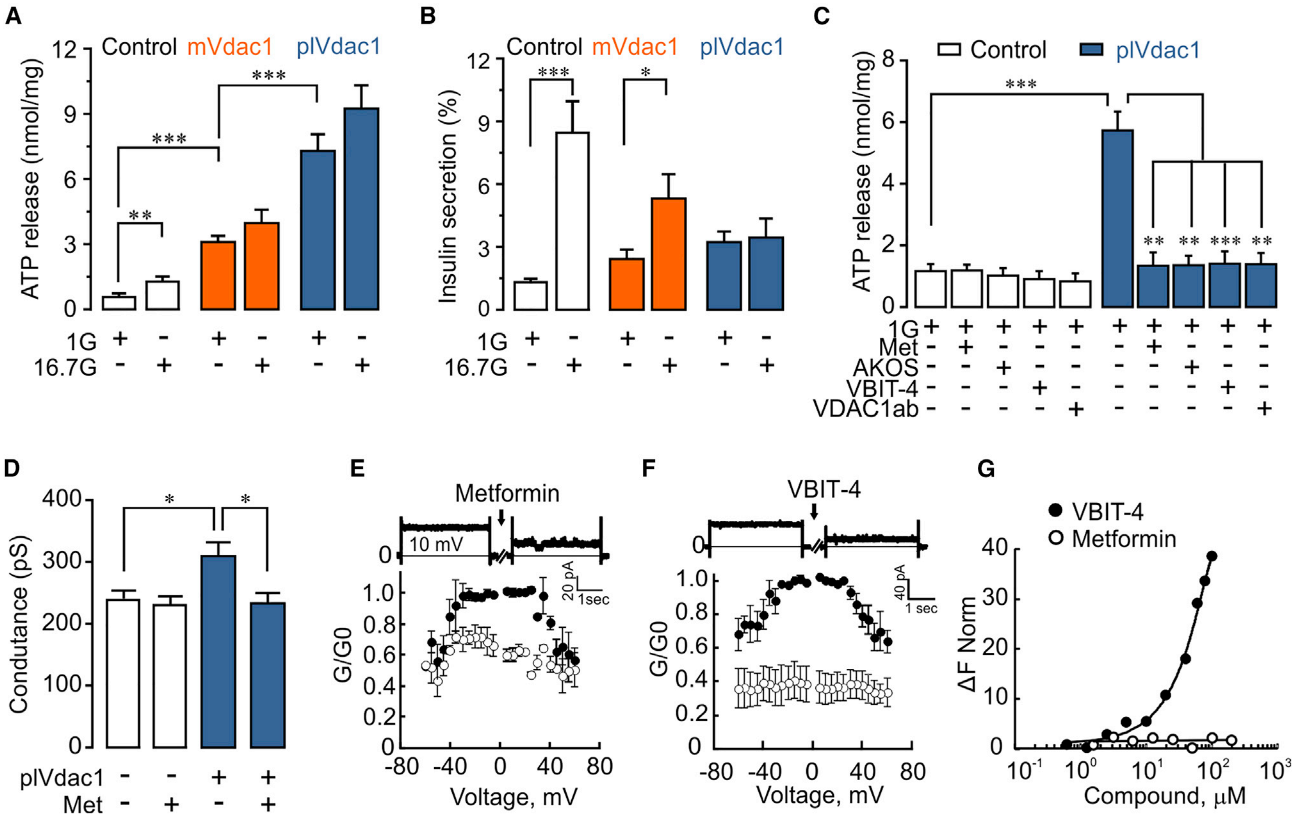

Figure 5. VDAC1 Cell Surface Expression Alters INS-1 Cell ATP Handling, Insulin Secretion, and Membrane Conductance

(A) ATP release after 1 hr incubation at 1 or 16.7 mM glucose from INS-1 cells transfected with either mitochondrial *Vdac1* (*mtVdac1*) or plasma membrane-targeted *Vdac1* (*plVdac1*) and control (empty plasmid). Mean ± SEM (n = 4).

(B) GSIS measured in the same experiments as in (A).

(C) Effect of VDAC1 antibody (VDAC1-ab, 10 nM), metformin, or the VDAC1 inhibitors AKOS022075291 and VBIT-4 (20 μM each) on ATP release after 1 hr exposure at 1 mM glucose of INS-1 cells transfected with control plasmid or *plVdac1*. Mean ± SEM from at least three independent experiments.

(D) Membrane conductance (whole-cell patch clamp) in control and *plVdac1*-transfected INS-1 cells in the presence or absence of metformin (20 μM) within 1 hr. Mean ± SEM of 15 cells in each group are shown.

(E) Metformin (30 μM) reduces conductance of VDAC1 reconstituted in planar lipid bilayers. Average steady-state conductance measured at the indicated voltage, before (●) and 10–30 min after metformin addition (○). Mean ± SEM of three independent measurements. A representative trace at 10 mV is shown at the top.

(F) Same as in (E) using VBIT-4 (20 μM).

(G) Interaction of VBIT-4 (●) or metformin (○) with soluble VDAC1 (162 nM), using microscale thermophoresis. Results are presented as percent of the bound fraction calculated as given in method.

INS-1 cells is abolished by the acute addition of either VDAC1 antibody (Figure S5D) or metformin (Figure 5D). Superfusion with metformin does not affect membrane currents in control INS-1 cells while abrogating the elevated conductance in plVdac1-transfected cells (Figure 5D).

As mtVDAC1 and pl-VDAC1 display identical amino acid sequences (Shoshan-Barmatz et al., 2010; Stadtmuller et al., 1999), we next performed channel conductance recordings with purified mitochondrial VDAC1 protein reconstituted into planar lipid bilayers (Ben-Hail et al., 2016). Metformin (30 μM) decreases channel conductance to a low conducting state imposed (Figures 5E, S5E, and S5F) like the specific VDAC inhibitor VBIT-4 (Figures 5F, S5G, and S5H). Nonetheless, metformin binding to VDAC1 might be voltage dependent, as 20 min preincubation with 100 μM metformin causes stronger current inhibition (at ±60 mV) in the isolated channel preparation (results not shown). These results demonstrate that metformin at low, therapeutic concentrations, directly inhibits VDAC1 conductance. To achieve quantitative analysis of the interaction of metformin with VDAC1 and extract a binding affinity coefficient (K_d) for the interaction we used microscale thermophoresis (MST). MST is based on changes in the diffusion of the complex protein-ligand. In contrast to VBIT-4, there is no apparent binding to VDAC1 of metformin (concentrations from 0.625 to 200 μM) (Figure 5G). Metformin inhibits the bilayer reconstituted-VDAC1 conductance but shows no apparent interaction with soluble protein. This may be due to failure of inducing changes in protein conformation detectable by thermophoresis. Furthermore, the acute effect of metformin on VDAC1 solute permeation in intact cells is not mediated via activation of AMP kinase (Foretz et al., 2014) or through an antioxidant effect (Figure S5I). VDAC1 is a target in cancer therapy (Shoshan-Barmatz et al., 2017) and

the chemopreventive action of metformin (Safe et al., 2018) may in part be exerted through the here reported VDAC1 inhibition.

VDAC1 Surface Expression Involves Its Two Cysteine Residues

Cell surface mistargeting of VDAC1 in T2D may involve post-translational modification of its two cysteine residues (cys127/232) (Aram et al., 2010; Piroli et al., 2016), although they are not important for VDAC1-induced apoptosis (Aram et al., 2010). Cysteine-depleted Vdac1 is much more efficiently overexpressed in INS-1 cells than mtVdac1, while the reciprocal suppression of Vdac2 observed after mtVDAC1 overexpression (*cf.* Figures S2A and S2B) is absent (Figures 6A and 6B). Despite the much higher expression, cysteine-depleted Vdac1 shows 50% less plasma membrane near localization than mtVdac1, as revealed by total internal reflection fluorescence microscopy (see Buda et al., 2013) (Figures 6C and 6D). Moreover, in contrast to mtVDAC1, the cellular ATP/ADP ratio and its increase by glucose stimulation are largely preserved (Figures 6E, 6F, and S6), as is cellular ATP content (Figure 6G). Furthermore, cysteine-depleted Vdac1-expressing cells do not display increased ATP release, which is very pronounced in mtVdac1 cells (Figure 6H). The preserved cellular ATP content and ATP generation by glucose explain the near normal GSIS in the cells transfected with the mutant VDAC1 (Figure 6I). These results are compatible with VDAC1 targeting to the β cell plasma membrane by post-translational cysteine modification, leading to ATP loss from the cells and impaired GSIS.

VDAC1 Surface Expression Causes Impaired GSIS in Islets from Diabetic Mice

Next, we investigated islet cells from diabetic db/db mice. Like islets from T2D donors, β cells from hyperglycemic *db/db*, but not from normoglycemic *C57/bl*, mice show surface expression of VDAC1 (Figures S7A and S7B). This is associated with increased Vdac1 mRNA (Figure S7C). ATP levels in islets from hyperglycemic *db/db* mice are not raised by 16.7 mM glucose and there is increased ATP release (Figures 7A and 7B). As expected from the VDAC1 surface localization, acute addition (1 hr) of VDAC1 antibody or metformin reduces ATP release and increases its cellular levels (Figures 7A and 7B). Consequently, ATP generation and GSIS are markedly enhanced when VDAC1 is inhibited during the high glucose stimulation (Figures 7A and 7C). Neither ATP content nor the stimulated GSIS is affected by VDAC1 inhibitors, including VBIT-4, in islets from normoglycemic *C57/bl* mice (Figures S7D and S7E), confirming that VDAC1 mistargeting is restricted to diabetic β cells.

Inhibition of VDAC1 Restores Insulin Secretion in T2D Donor Islets

Subsequently, we investigated whether inhibiting cell membrane VDAC1 conductivity also could ameliorate β cell function in human islets. Inhibition of VDAC1 using metformin, VDAC1 inhibitors, or antibodies does not alter ATP content and cellular reductive capacity after culture at 5 mM glucose, but markedly improves metabolism during glucotoxic conditions or in T2D islets (Figures S7F and S7G). Inclusion of VDAC1 antibody or metformin in the culture medium, while not affecting secretion after culture at 5 mM glucose, prevents the attenuation of GSIS observed in the islets cultured at 20 mM glucose (Figure 7D). Similar results were previously reported with metformin in human islets under glucotoxic conditions (Masini et al., 2014) and the drug improved the function of T2D islets (Marchetti et al., 2004). To probe for membrane effects of VDAC1 antibody, metformin, or VBIT-4, we pooled results of islets from five T2D donors and one with IGT. After only 1 hr exposure to the compounds, VDAC1 inhibition increases islet ATP content both at 1 and 16.7 mM glucose. Notably, the ATP-increasing effect of glucose is 5-fold enhanced by VDAC1 inhibition (Figure 7E). The T2D islets display severe blunting of GSIS. Remarkably, GSIS is increased nearly 4-fold in parallel with the improved ATP generation by each of the four VDAC1 inhibitors (Figure 7F; cf. 2I and 7D for GSIS in ND islets). Of note, acute addition of VBIT-4 concentrations up to 100 μM does not affect GSIS in ND islets (Figure S7H). Thus, GSIS is markedly improved in T2D islets by the acute inhibition of plVDAC1, suggesting that VDAC1 mistargeting rather than the decreased VDAC2 gene expression (cf. Figures 1A–1E) underlies the impaired β cell function. It cannot be excluded that the decreased VDAC2 expression or reduced β cell mass (Marselli et al., 2014; Meier and Bonadonna, 2013) in the T2D islets underlies the incomplete restoration of GSIS.

Inhibition of VDAC1 Prevents Hyperglycemia in db/db Mice

The encouraging results in T2D islets prompted us to study the effects of the VDAC1 inhibitor VBIT-4 *in vivo*. To this end, we subjected diabetes-prone young *db/db* mice to daily intraperitoneal injections of VBIT-4 for 5 weeks, starting at age 42 days. This treatment is not affecting blood glucose in control *C57/bl* mice but prevents the development of the severe hyperglycemia, seen in vehicle-injected *db/db* mice. Upon VBIT-4 cessation, blood glucose concentrations increase gradually over several weeks, reaching those of vehicle-treated animals (Figure 7G). There is no effect on body weight (Figure S7I). The drug markedly improves glucose tolerance and GSIS *in vivo* (Figures 7H–7J and S7J). Insulin sensitivity was also improved in the treated *db/db* mice as calculated by the homeostasis model assessment of insulin resistance (HOMA-IR) (Bowe et al., 2014) (Figure S7K), which may reflect involvement of altered VDAC1 expression in insulin target tissues (Li et al., 2016; Sasaki et al., 2012). GSIS was also normalized in islets isolated from VBIT-4-treated *db/db* mice (Figure S7L). The early administration of VBIT-4 also prevents increases in water consumption and polyuria in the *db/db* mice, but only marginally lowers blood glucose in 14-week-old animals with manifest hyperglycemia (data not shown). It is noteworthy, that VBIT-4 appears to be more efficient than metformin administered before the outbreak of diabetes in the same animal model (Cao et al., 2014). A possible explanation is that metformin inhibits VDAC1 conductance less efficiently than VBIT-4. Nonetheless, both metformin and VBIT-4 also counteracted *VDAC1* overexpression, preventing mistargeting to the β cell surface under glucotoxic conditions (see scheme in Figure S7M). Collectively, early initiation of therapy with a VDAC1 inhibitor blocks the development of diabetes in this T2D mouse model. Through the prevention of VDAC1 gene expression, VBIT-4 may have disease-modifying actions. We base this on the remarkable delay (at least 3 weeks) in the

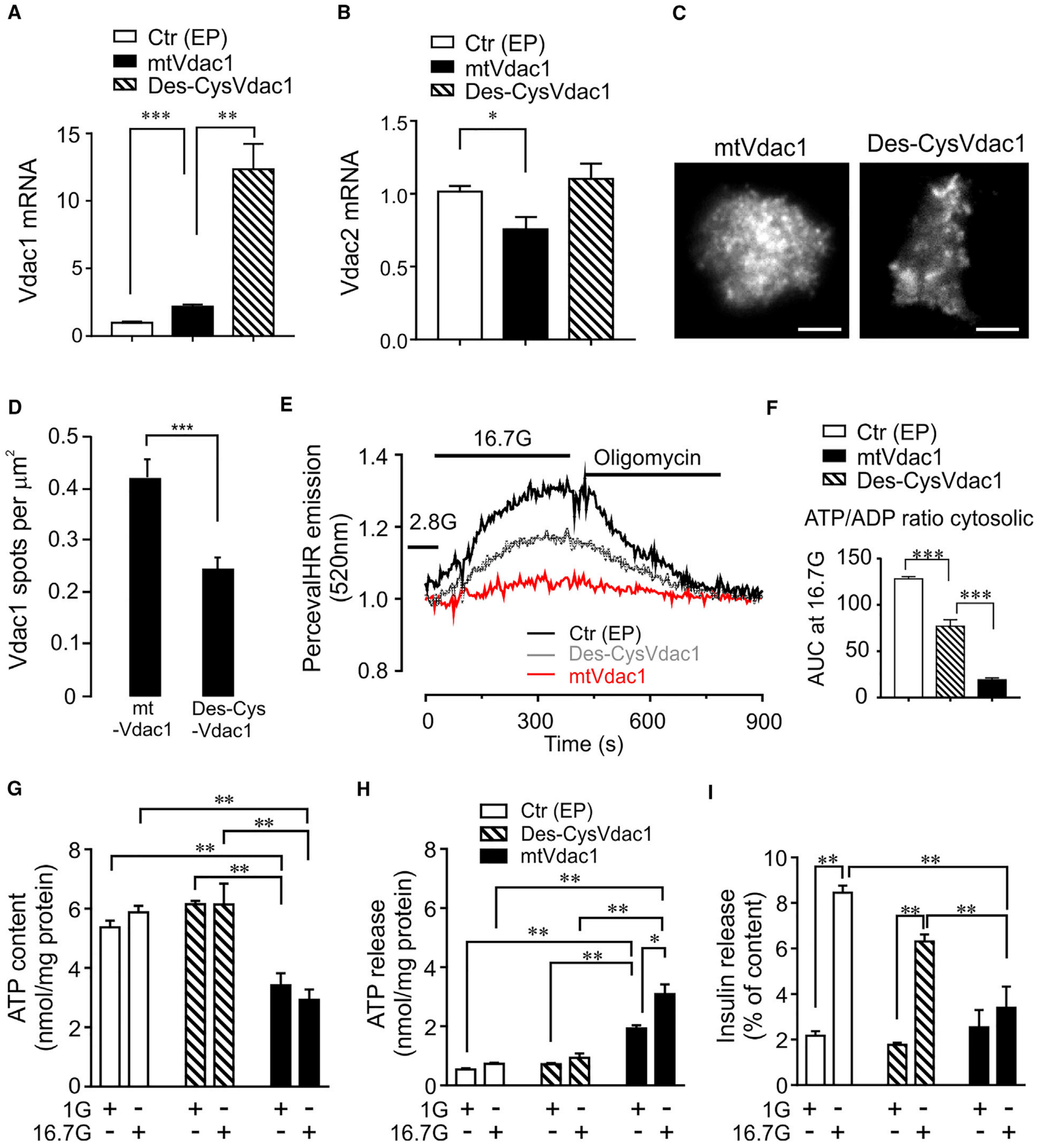

Figure 6. Overexpression of desCys(127/232)VDAC1 (Des-CysVDAC1) Is Less Harmful for INS-1 Cell Function than mtVDAC1

(A and B) Vdac1 mRNA expression (A) and Vdac2 mRNA expression (B) in cells transfected with *mtVdac1* or Des-*CysVdac1*. Results are mean ± SEM of four independent experiments with 1–4 replicates in each.

(C) Representative image of plasma membrane-near localization of mtVdac1 or Des-CysVdac1 in INS-1 cells assessed by total internal reflection fluorescence microscopy. Scale bar indicates 5 μm.

(D) Surface density of *mtVdac1* and Des-*CysVdac1* in INS-1 cells; Mean ± SEM of 30 cells each from three independent experiments.

(E) Cytosolic ATP/ADP ratio measured in single INS-1 cells (Ex/Em 488/520, 37°C) by confocal microscopy after co-transfection with PercevalHR and either *mtVdac1* or Des-*CysVdac1*.

(F) Glucose-induced increases in cytosolic ATP/ADP ratio are largely preserved in des-*Vdac1* and abolished in *mtVdac1* overexpressing INS-1 cells. AUC for glucose stimulation of five to ten analyzed cells from six different experiments, for AUC of the values after oligomycin addition (see Figure S6).

(G–I) ATP content (G), ATP release (H), and insulin release (I) from INS-1 cells transfected with empty plasmid (Ctr), Des-CysVDAC1, or mtVDAC1 plasmids and incubated at 1 mM (1G) or 16.7 mM glucose (16.7G) for 1 hr (n = 5).

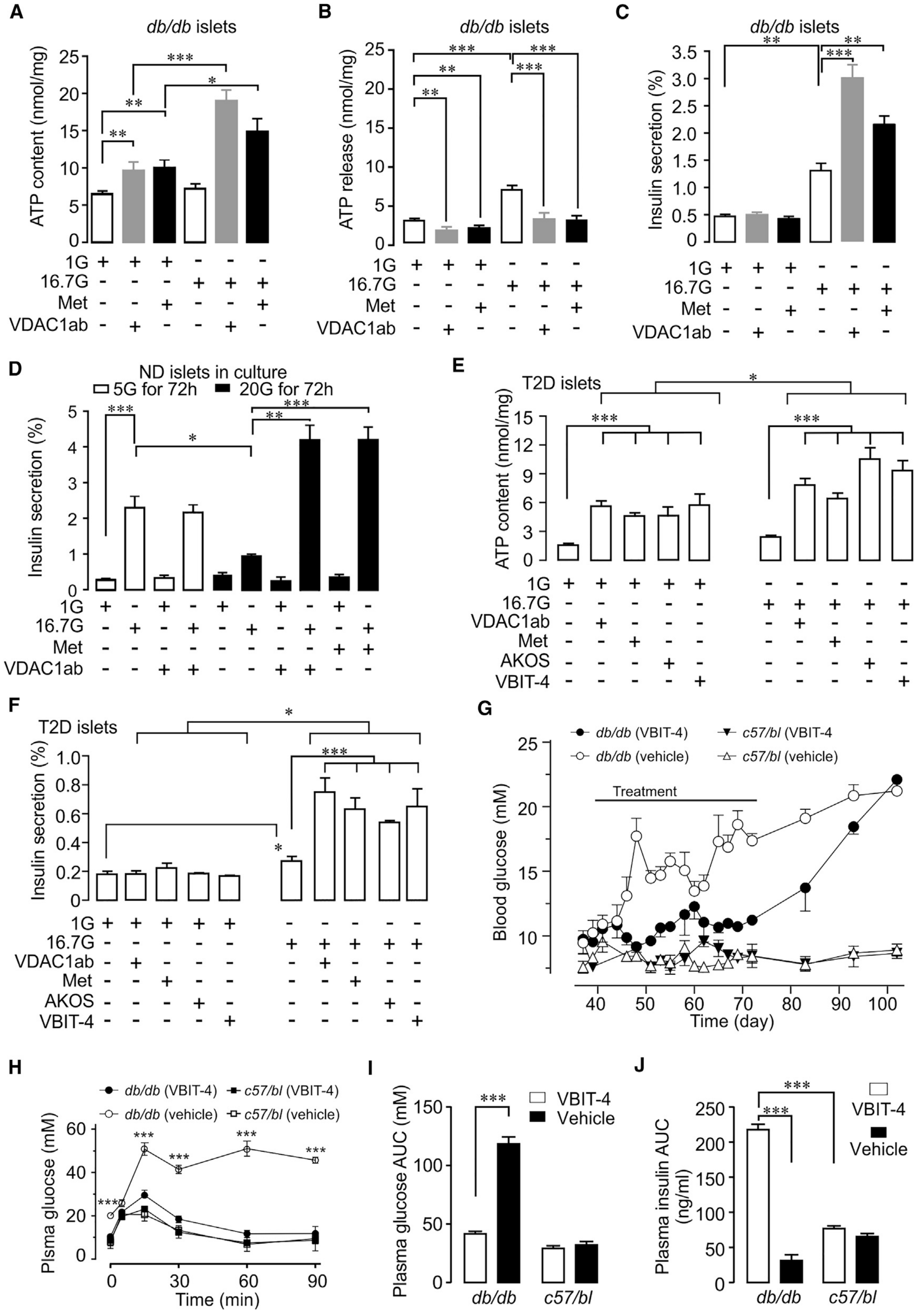

Figure 7. Inhibition of Cell Surface Mistargeted VDAC1 Restores GSIS in Prediabetic *db/db* Mice and in Human T2D Islets

(A–C) One-hour exposure to VDAC1 antibody (10 nM) or metformin (20 μM) restores impaired glucose-stimulated ATP generation in islets of diabetic *db/db* mice in parallel with suppression of ATP release (B) and augments GSIS (C). Mean ± SEM (n = 4).

(legend continued on next page)

establishment of severe hyperglycemia after discontinuation of the compound.

Conclusion

We demonstrate that the harmful effects of glucose cause *VDAC1* induction in T2D. This novel diabetes executer protein impairs β cell metabolism-secretion coupling through its mistargeting to the β cell membrane with consequent ATP depletion (Figure S7M). Our results suggest VDAC1 as a novel target in diabetes therapy with the potential to prevent progression of prediabetes to overt T2D. We also report that metformin inhibits VDAC1 function, a novel mechanism of action for the antidiabetic drug. The β cell protective action of metformin with ameliorated insulin secretion rather than improved insulin effectiveness was recently reported in T2D subjects (Retnakaran et al., 2018). It is likely that this beneficial effect targets VDAC1 gene expression and function in the β cells.

Limitations of Study

The main limitations of the current study are the limited experiments in diabetic animal models. In particular, in-depth analysis of the relative importance of VDAC1 inhibition for the amelioration of insulin secretion from the β cells relative to action on insulin target tissues, such as liver, muscle, and fat, needs to be performed. It would also be important to crossbreed diabetic mice with Vdac1-KO mice. This is, however, difficult due to the reduced fertility of Vdac1-KO mice (Raghavan et al., 2012). The reciprocal regulation of gene expression between VDAC1 and VDAC2, which we have reported, requires further investigation both *in vitro* and *in vivo*. However, such *in vivo* studies would require β cell-targeted Vdac1 and Vdac2 deletion, as global Vdac2-KO mice are embryonic lethal (Raghavan et al., 2012).

STAR★METHODS

Detailed methods are provided in the online version of this paper and include the following:

- KEY RESOURCES TABLE
- CONTACT FOR REAGENT AND RESOURCE SHARING
- EXPERIMENTAL MODEL AND SUBJECT DETAILS
  - Cell line
  - Human Islet Preparations
  - Mouse Islet Preparations
- METHOD DETAILS
  - Cell Culture
  - Plasmid and Transient Transfections
  - Histone Acetyltransferase p300 (Ep300) Knock-out Cells
  - Insulin Secretion in Cultured INS-1 Cells
  - Conductance Measurement by Patch Clamp Recording
  - VDAC Current Measurement on the Reconstituted VDAC1 in Planar Lipid Bilayer (PLB)
  - VDAC1 Binding Study
  - Intraperitoneal Glucose Tolerance Tests (IPGTT)
  - Glucose-stimulated Insulin Secretion (GSIS) in Human and Mouse Islets
  - Quantitative Polymerase Chain Reaction (qPCR)
  - Immunoblotting
  - Immunostaining and Confocal Imaging
  - Single Cell ATP/ADP Ratio Measurement
  - TIRF Microscopy
  - Small Interfering RNA (siRNA) for Protein Expression Silencing
  - Silencing by shRNA Mediated (Lentivirus) in Human Islets
  - Measurement of Cellular Reductive Capacity (MTS), Apoptosis and Viability
  - Measurement of Oxygen Consumption Rate (OCR)
  - ATP Determination
  - Mitochondrial and Cytosolic Ca^{2+} Imaging in Single Cells
- QUANTIFICATION AND STATISTICAL ANALYSIS
- DATA AND SOFTWARE AVAILABILITY

SUPPLEMENTAL INFORMATION

Supplemental Information includes seven figures and two tables and can be found with this article online at https://doi.org/10.1016/j.cmet.2018.09.008.

ACKNOWLEDGMENTS

We would like to thank Britt-Marie Nilsson and Anna Maria Ramsay for their skillful and dedicated technical assistance. We are grateful to Dr. Ola Hansson, Dr. Joao Fadista, and Dr. Petter Storm for help with gene analysis. We are indebted to Dr. Ruchi Jain for the calculation of the HOMA-IR results. We also thank Prof. Michele Solimena, Dr. Nils Wierup, and Dr. Andreas Lindquist for immunohistochemical analysis. This study was supported by grants to A. Salehi from Diabetes & Wellness Foundation Sweden, Öresund Diabetes Academy, Mats Paulsson Foundation, Forget foundation, Bo and Kerstin Hjelt foundation for diabetes type 2, and Lund University innovation board. A grant to E.Z. from the Crafoord Foundation, a grant to V.S.-B. from Israel Science Foundation, and Sima and Philip Needleman research funds are appreciated; grants to L.G. were from the Swedish Research Council and an ERC Advanced Researcher grant (GA269045); and E.Z.'s position is covered by a project grant

(D) Insulin secretion in human ND islets cultured at 5 or 20 mM glucose (72 hr) in the presence and absence of VDAC1 antibody or metformin, followed by 1 hr incubation at 1G or 16.7G.

(E) Acute addition of VDAC1 inhibitors (1 hr) improves glucose-stimulated ATP generation in islets from T2D donors.

(F) Improved GSIS in the T2D islets shown in (E). Mean ± SEM (3–6 donors).

(G) The VDAC1 inhibitor VBIT-4 prevents hyperglycemia in prediabetic *db/db* mice injected at 6 weeks (25 mg/kg daily intraperitoneally [i.p.]) compared with vehicle-treated *db/db* mice (n = 12). *C57/bl6* mice receiving either VBIT-4 (n = 5) or vehicle (n = 6) are also depicted. Six *db/db* mice from each group were followed for another 3–4 weeks for reversibility of the treatment. All *C57/bl6* mice were monitored throughout. Note the gradual increase and long duration before reaching full hyperglycemia after VBIT-4 cessation.

(H) Plasma glucose concentrations during intraperitoneal glucose tolerance test (2 g/kg) in *db/db* or *C57/bl6* mice after VBIT-4 treatment as in (G). Mean ± SEM of 12 mice (12 *db/db* and 5–6 *C57/bl6* in each group).

(I) AUC for plasma glucose.

(J) AUC for plasma insulin (for plasma insulin values, see Figure S7J).

from the Knut and Alice Wallenberg Foundation (to E.R.). Grants to E.R. were from the Swedish Research Council, the Swedish Diabetes Association, Diabetes Wellness Sweden Foundation, and grants for clinical Research (ALF). The study was also supported by the Swedish Research Council, Linnaeus grant (Dnr 349-2006-237); Strategic Research Area Exodiab (Dnr 2009-1039); and Swedish Foundation for Strategic Research LUDC-IRC (Dnr IRC15-0067).

AUTHOR CONTRIBUTIONS

A. Salehi and C.B.W. designed the study and supervised the project, conducted data analysis, and wrote the manuscript with feedback from all authors. A. Salehi also conducted experiments on isolated islets and INS-1 cells. E.Z. conducted experiments, imaging, and data analysis. I.M.A.-A. conducted the experiments on human islets, transfected INS-1 cells, and ran qPCR and insulin secretion analysis. S.M. and A. Soni performed qPCR, western blots, and insulin secretion experiments. N.V. performed a later supervised cell imaging of the ATP/ADP ratio. C.L., D.B.-H., and Y.Y. conducted electrophysiology and apoptosis experiments. O.A. analyzed RNA sequencing and gene expression. M.A. contributed to design of qPCR primers and immunoblot experiments. P.B. and Y.D.M. performed experiments on INS-1 (*Ep300*$^{-/-}$) cells. L.G. and E.R. designed and supervised RNA sequencing and electrophysiology, respectively, and edited the manuscript. V.S.-B. supervised experiments on isolated VDAC1 protein. All authors revised and approved the manuscript.

DECLARATION OF INTERESTS

A. Salehi and C.B.W. are founders of Abarceo Pharma, Malmö, Sweden, and are members of its board. They are also named as inventors on a patent regarding VDAC1 as a drug target in T2D.

Received: February 28, 2018
Revised: July 12, 2018
Accepted: September 8, 2018
Published: October 4, 2018

REFERENCES

Akanda, N., Tofighi, R., Brask, J., Tamm, C., Elinder, F., and Ceccatelli, S. (2008). Voltage-dependent anion channels (VDAC) in the plasma membrane play a critical role in apoptosis in differentiated hippocampal neurons but not in neural stem cells. Cell Cycle *7*, 3225–3234.

Ahmed, M., Muhammed, S.J., Kessler, B., and Salehi, A. (2010). Mitochondrial proteome analysis reveals altered expression of voltage dependent anion channels in pancreatic beta-cells exposed to high glucose. Islets *2*, 283–292.

Anello, M., Lupi, R., Spampinato, D., Piro, S., Masini, M., Boggi, U., Del Prato, S., Rabuazzo, A.M., Purrello, F., and Marchetti, P. (2005). Functional and morphological alterations of mitochondria in pancreatic beta cells from type 2 diabetic patients. Diabetologia *48*, 282–289.

Aram, L., Geula, S., Arbel, N., and Shoshan-Barmatz, V. (2010). VDAC1 cysteine residues: topology and function in channel activity and apoptosis. Biochem. J. *427*, 445–454.

Aroda, V.R., Knowler, W.C., Crandall, J.P., Perreault, L., Edelstein, S.L., Jeffries, S.L., Molitch, M.E., Pi-Sunyer, X., Darwin, C., Heckman-Stoddard, B.M., et al. (2017). Metformin for diabetes prevention: insights gained from the diabetes prevention program/diabetes prevention program outcomes study. Diabetologia *60*, 1601–1611.

Ben-Hail, D., Begas-Shvartz, R., Shalev, M., Shteinfer-Kuzmine, A., Gruzman, A., Reina, S., De Pinto, V., and Shoshan-Barmatz, V. (2016). Novel compounds targeting the mitochondrial protein VDAC1 inhibit apoptosis and protect against mitochondria dysfunction. J. Biol. Chem. *291*, 24986–25003.

Blodgett, D.M., Nowosielska, A., Afik, S., Pechhold, S., Cura, A.J., Kennedy, N.J., Kim, S., Kucukural, A., Davis, R.J., Kent, S.C., et al. (2015). Novel observations from next-generation RNA sequencing of highly purified human adult and fetal islet cell subsets. Diabetes *64*, 3172–3181.

Boden, G., Ruiz, J., Kim, C.J., and Chen, X. (1996). Effects of prolonged glucose infusion on insulin secretion, clearance, and action in normal subjects. Am. J. Physiol. *270*, E251–E258.

Bompada, P., Atac, D., Luan, C., Andersson, R., Omella, J.D., Laakso, E.O., Wright, J., Groop, L., and De Marinis, Y. (2016). Histone acetylation of glucose-induced thioredoxin-interacting protein gene expression in pancreatic islets. Int. J. Biochem. Cell Biol. *81*, 82–91.

Bowe, J.E., Franklin, Z.J., Hauge-Evans, A.C., King, A.J., Persaud, S.J., and Jones, P.M. (2014). Metabolic phenotyping guidelines: assessing glucose homeostasis in rodent models. J. Endocrinol. *222*, G13–G25.

Buda, P., Reinbothe, T., Nagaraj, V., Mahdi, T., Luan, C., Tang, Y., Axelsson, A.S., Li, D., Rosengren, A.H., Renstrom, E., et al. (2013). Eukaryotic translation initiation factor 3 subunit e controls intracellular calcium homeostasis by regulation of cav1.2 surface expression. PLoS One *8*, e64462.

Buettner, R., Papoutsoglou, G., Scemes, E., Spray, D.C., and Dermietzel, R. (2000). Evidence for secretory pathway localization of a voltage-dependent anion channel isoform. Proc. Natl. Acad. Sci. USA *97*, 3201–3206.

Cao, K., Xu, J., Zou, X., Li, Y., Chen, C., Zheng, A., Li, H., Li, H., Szeto, I.M., Shi, Y., et al. (2014). Hydroxytyrosol prevents diet-induced metabolic syndrome and attenuates mitochondrial abnormalities in obese mice. Free Radic. Biol. Med. *67*, 396–407.

Cha-Molstad, H., Saxena, G., Chen, J., and Shalev, A. (2009). Glucose-stimulated expression of Txnip is mediated by carbohydrate response element-binding protein, p300, and histone H4 acetylation in pancreatic beta cells. J. Biol. Chem. *284*, 16898–16905.

Clark, K., MacKenzie, K.F., Petkevicius, K., Kristariyanto, Y., Zhang, J., Choi, H.G., Peggie, M., Plater, L., Pedrioli, P.G., McIver, E., et al. (2012). Phosphorylation of CRTC3 by the salt-inducible kinases controls the interconversion of classically activated and regulatory macrophages. Proc. Natl. Acad. Sci. USA *109*, 16986–16991.

De Pinto, V., Messina, A., Lane, D.J., and Lawen, A. (2010). Voltage-dependent anion-selective channel (VDAC) in the plasma membrane. FEBS Lett. *584*, 1793–1799.

Doliba, N.M., Qin, W., Najafi, H., Liu, C., Buettger, C.W., Sotiris, J., Collins, H.W., Li, C., Stanley, C.A., Wilson, D.F., et al. (2012). Glucokinase activation repairs defective bioenergetics of islets of Langerhans isolated from type 2 diabetics. Am. J. Physiol. Endocrinol. Metab. *302*, E87–E102.

Fadista, J., Vikman, P., Laakso, E.O., Mollet, I.G., Esguerra, J.L., Taneera, J., Storm, P., Osmark, P., Ladenvall, C., Prasad, R.B., et al. (2014). Global genomic and transcriptomic analysis of human pancreatic islets reveals novel genes influencing glucose metabolism. Proc. Natl. Acad. Sci. USA *111*, 13924–13929.

Fernandez-Echevarria, C., Diaz, M., Ferrer, I., Canerina-Amaro, A., and Marin, R. (2014). Abeta promotes VDAC1 channel dephosphorylation in neuronal lipid rafts. Relevance to the mechanisms of neurotoxicity in Alzheimer's disease. Neuroscience *278*, 354–366.

Foretz, M., Guigas, B., Bertrand, L., Pollak, M., and Viollet, B. (2014). Metformin: from mechanisms of action to therapies. Cell Metabol. *20*, 953–966.

Janjic, D., and Wollheim, C.B. (1992). Islet cell metabolism is reflected by the MTT (tetrazolium) colorimetric assay. Diabetologia *35*, 482–485.

Li, X., Kover, K.L., Heruth, D.P., Watkins, D.J., Moore, W.V., Jackson, K., Zang, M., Clements, M.A., and Yan, Y. (2015). New insight into metformin action: regulation of ChREBP and FOXO1 activities in endothelial cells. Mol. Endocrinol. *29*, 1184–1194.

Li, X., Wang, H., Yao, B., Xu, W., Chen, J., and Zhou, X. (2016). lncRNA H19/miR-675 axis regulates cardiomyocyte apoptosis by targeting VDAC1 in diabetic cardiomyopathy. Sci. Rep. *6*, 36340.

Ligthart, S., van Herpt, T.T., Leening, M.J., Kavousi, M., Hofman, A., Stricker, B.H., van Hoek, M., Sijbrands, E.J., Franco, O.H., and Dehghan, A. (2016). Lifetime risk of developing impaired glucose metabolism and eventual progression from prediabetes to type 2 diabetes: a prospective cohort study. Lancet Diabetes Endocrinol. *4*, 44–51.

Lyssenko, V., Jonsson, A., Almgren, P., Pulizzi, N., Isomaa, B., Tuomi, T., Berglund, G., Altshuler, D., Nilsson, P., and Groop, L. (2008). Clinical risk factors, DNA variants, and the development of type 2 diabetes. N. Engl. J. Med. *359*, 2220–2232.

MacDonald, M.J., Longacre, M.J., Langberg, E.C., Tibell, A., Kendrick, M.A., Fukao, T., and Ostenson, C.G. (2009). Decreased levels of metabolic enzymes in pancreatic islets of patients with type 2 diabetes. Diabetologia *52*, 1087–1091.

Marchetti, P., Del Guerra, S., Marselli, L., Lupi, R., Masini, M., Pollera, M., Bugliani, M., Boggi, U., Vistoli, F., Mosca, F., et al. (2004). Pancreatic islets from type 2 diabetic patients have functional defects and increased apoptosis that are ameliorated by metformin. J. Clin. Endocrinol. Metab. *89*, 5535–5541.

Marselli, L., Suleiman, M., Masini, M., Campani, D., Bugliani, M., Syed, F., Martino, L., Focosi, D., Scatena, F., Olimpico, F., et al. (2014). Are we overestimating the loss of beta cells in type 2 diabetes? Diabetologia *57*, 362–365.

Masini, M., Anello, M., Bugliani, M., Marselli, L., Filipponi, F., Boggi, U., Purrello, F., Occhipinti, M., Martino, L., Marchetti, P., et al. (2014). Prevention by metformin of alterations induced by chronic exposure to high glucose in human islet beta cells is associated with preserved ATP/ADP ratio. Diabetes Res. Clin. Pract. *104*, 163–170.

Meier, J.J., and Bonadonna, R.C. (2013). Role of reduced beta-cell mass versus impaired beta-cell function in the pathogenesis of type 2 diabetes. Diabetes Care *36* (*Suppl 2*), S113–S119.

Naghdi, S., and Hajnoczky, G. (2016). VDAC2-specific cellular functions and the underlying structure. Biochim. Biophys. Acta *1863*, 2503–2514.

Nilsson, E., and Ling, C. (2017). DNA methylation links genetics, fetal environment, and an unhealthy lifestyle to the development of type 2 diabetes. Clin. Epigenetics *9*, 105.

Okada, S.F., O'Neal, W.K., Huang, P., Nicholas, R.A., Ostrowski, L.E., Craigen, W.J., Lazarowski, E.R., and Boucher, R.C. (2004). Voltage-dependent anion channel-1 (VDAC-1) contributes to ATP release and cell volume regulation in murine cells. J. Gen. Physiol. *124*, 513–526.

Palanisamy, S., Yien, E.L.H., Shi, L.W., Si, L.Y., Qi, S.H., Ling, L.S.C., Lun, T.W., and Chen, Y.N. (2018). Systematic review of efficacy and safety of newer antidiabetic drugs approved from 2013 to 2017 in controlling HbA1c in diabetes patients. Pharmacy (Basel) *6*, https://doi.org/10.3390/pharmacy6030057.

Piroli, G.G., Manuel, A.M., Clapper, A.C., Walla, M.D., Baatz, J.E., Palmiter, R.D., Quintana, A., and Frizzell, N. (2016). Succination is increased on select proteins in the brainstem of the NADH dehydrogenase (ubiquinone) Fe-S protein 4 (Ndufs4) knockout mouse, a model of leigh syndrome. Mol. Cell. Proteomics *15*, 445–461.

Poungvarin, N., Lee, J.K., Yechoor, V.K., Li, M.V., Assavapokee, T., Suksaranjit, P., Thepsongwajja, J.J., Saha, P.K., Oka, K., and Chan, L. (2012). Carbohydrate response element-binding protein (ChREBP) plays a pivotal role in beta cell glucotoxicity. Diabetologia *55*, 1783–1796.

Raghavan, A., Sheiko, T., Graham, B.H., and Craigen, W.J. (2012). Voltage-dependent anion channels: novel insights into isoform function through genetic models. Biochim. Biophys. Acta *1818*, 1477–1485.

Retnakaran, R., Choi, H., Ye, C., Kramer, C.K., and Zinman, B. (2018). Two-year trial of intermittent insulin therapy vs metformin for the preservation of beta-cell function after initial short-term intensive insulin induction in early type 2 diabetes. Diabetes Obes. Metab. *20*, 1399–1407.

Ribe, E.M., and Lovestone, S. (2016). Insulin signalling in Alzheimer's disease and diabetes: from epidemiology to molecular links. J. Int. Med. *280*, 430–442.

Safe, S., Nair, V., and Karki, K. (2018). Metformin-induced anticancer activities: recent insights. Biol. Chem. *399*, 321–335.

Sasaki, K., Donthamsetty, R., Heldak, M., Cho, Y.E., Scott, B.T., and Makino, A. (2012). VDAC: old protein with new roles in diabetes. Am. J. Physiol. Cell Physiol. *303*, C1055–C1060.

Shaked, M., Ketzinel-Gilad, M., Cerasi, E., Kaiser, N., and Leibowitz, G. (2011). AMP-activated protein kinase (AMPK) mediates nutrient regulation of thioredoxin-interacting protein (TXNIP) in pancreatic beta-cells. PLoS One *6*, e28804.

Shalev, A. (2014). Minireview: thioredoxin-interacting protein: regulation and function in the pancreatic beta-cell. Mol. Endocrinol. *28*, 1211–1220.

Shoshan-Barmatz, V., De Pinto, V., Zweckstetter, M., Raviv, Z., Keinan, N., and Arbel, N. (2010). VDAC, a multi-functional mitochondrial protein regulating cell life and death. Mol. Aspects Med. *31*, 227–285.

Shoshan-Barmatz, V., Krelin, Y., Shteinfer-Kuzmine, A., and Arif, T. (2017). Voltage-dependent anion channel 1 as an emerging drug target for novel anti-cancer therapeutics. Front. Oncol. *7*, 154.

Smilansky, A., Dangoor, L., Nakdimon, I., Ben-Hail, D., Mizrachi, D., and Shoshan-Barmatz, V. (2015). The voltage-dependent anion channel 1 mediates amyloid beta toxicity and represents a potential target for Alzheimer disease therapy. J. Biol. Chem. *290*, 30670–30683.

Stadtmuller, U., Eben-Brunnen, J., Schmid, A., Hesse, D., Klebert, S., Kratzin, H.D., Hesse, J., Zimmermann, B., Reymann, S., Thinnes, F.P., et al. (1999). Mitochondria-derived and extra-mitochondrial human type-1 porin are identical as revealed by amino acid sequencing and electrophysiological characterisation. Biol. Chem. *380*, 1461–1466.

Tantama, M., and Yellen, G. (2014). Imaging changes in the cytosolic ATP-to-ADP ratio. Methods Enzymol. *547*, 355–371.

Thinnes, F.P. (2015). After all, plasmalemmal expression of type-1 VDAC can be understood. Phosphorylation, nitrosylation, and channel modulators work together in vertebrate cell volume regulation and either apoptotic pathway. Front. Physiol. *6*, 126.

Thivolet, C., Vial, G., Cassel, R., Rieusset, J., and Madec, A.M. (2017). Reduction of endoplasmic reticulum-mitochondria interactions in beta cells from patients with type 2 diabetes. PLoS One *12*, e0182027.

Wajchenberg, B.L. (2007). beta-Cell failure in diabetes and preservation by clinical treatment. Endocr. Rev. *28*, 187–218.

Weir, G.C., and Bonner-Weir, S. (2004). Five stages of evolving beta-cell dysfunction during progression to diabetes. Diabetes *53* (*Suppl 3*), S16–S21.

Wiederkehr, A., Szanda, G., Akhmedov, D., Mataki, C., Heizmann, C.W., Schoonjans, K., Pozzan, T., Spat, A., and Wollheim, C.B. (2011). Mitochondrial matrix calcium is an activating signal for hormone secretion. Cell Metab. *13*, 601–611.

Wiederkehr, A., and Wollheim, C.B. (2012). Mitochondrial signals drive insulin secretion in the pancreatic beta-cell. Mol. Cell Endocrinol. *353*, 128–137.

STAR★METHODS

KEY RESOURCES TABLE

REAGENT or RESOURCE	SOURCE	IDENTIFIER
Antibodies		
monoclonal mouse and rabbit anti-VDAC1 antibody (N-terminal)	Calbiochem	RRID: AB_2564843
monoclonal rabbit anti-VDAC1 antibody (N-terminal	abcam	RRID: AB_2687466
goat polyclonal anti-VDAC2	abcam	RRID: AB_778790
HRP-conjugated antigoat IgG	Abcam	RRID: AB_955424
HRP-conjugated anti-rabbit IgG	Bio-Rad	RRID: AB_11125757
Guinea pig polyclonal antibody to Insulin	Progen Biotechnic GmbH	RRID: AB_1542133
SNAP-25 antibody	SYSY	RRID: AB_887790
β-actin antibody	Sigma-Aldrich	RRID: AB_476744
Biological Samples		
Human islets and pancreatic sections	Nordic Network for Clinical Islet Transplantation	Professor Olle Korsgren, Uppsala University, Sweden
Mouse islets	In house isolation procedure	For information contact Dr A. Salehi
Chemicals, Peptides, and Recombinant Proteins		
siRNAs of VDAC1	Ambion	ASO0LYL9, ASO0LYL8, ASO0LYL7, ASO0LYL6,
siRNAs of VDAC2	Ambion	ASO0LYLC, ASO0LYLD, ASO0LYLE, ASO0LYLF
siRNAs of ChREBP	Ambion	ASO0NLVV, ASO0NLVW, ASO0NLVU
siRNAs of Txnip	Ambion	ASO0NLVR, ASO0NLVS, ASO0NLVT,
Human VDAC1 lentivirus shRNA	Santa Cruz Biotechnology	Sc-42355-V
Human VDAC2 lentivirus shRNA	Santa Cruz Biotechnology	Sc-42357-V
Polybrene	Santa Cruz Biotechnology	Sc-134220
VDAC1 plasmid construct	Source BioScience imagenes,	pDEST26
Plasma membrane lead mic-targeted VDAC1 (plVDAC1)	Albert Einstein College of Medicine, NY, USA	Buettner et al., 2000
Mitochondrial VDAC1 (mtVDAC1) (mic-targeted)	Albert Einstein College of Medicine, NY, USA	Buettner et al., 2000
DesCys(127/232)VDAC1 plasmid	Ben-Gurion University Negev, Beer-Sheva Israel	Aram et al., 2010
PercevalHR	Addgene	49,082
The multiple kinase inhibitor (MRT1)	In-house-made	Clark et al., 2012
VBIT-4 and AKOS-02	In-house-made	Ben-Hail et al., 2016
Resveratrol	AK Scientific	170134-5G
Metformin	Sigma-Aldrich	D150959-5G
N-Acetyl cysteine	Sigma-Aldrich	A7250-5G
2-Deoxy-D-glucose	Sigma-Aldrich	D8375-1G
Bovine serum albumin (fatty acid free)	Boehringer, Germany	N/A
Rotenone	Sigma-Aldrich	R8875-1G
Oligomycin	Calbiochem	1404-19-9
FCCP	Sigma-Aldrich	C2920-10MG
Rhod-2 AM	Invitrogen	R1245MP
Fluo-5F AM	Invitrogen	F14222

(*Continued on next page*)

Continued		
REAGENT or RESOURCE	SOURCE	IDENTIFIER
Deposited Data		
Data	This paper	GEO: GSE108072
Experimental Models: Organisms/Strains		
db/db mice	Janvier Laboratory, France	B6/KS genetic background
C57Bl mice	Janvier Laboratory, France	C57BL/6JRj
Oligonucleotides		
For the full list of Primers see Table S2	This paper	N/A
Human insulin ELISA	Mercodia	Cat# 10-1113
Rat Insulin ELISA	Mercodia	Cat# 10-1145
ATP Assay Kit (C/F)	BioVision	K354-100
MTS Kit	Promega	G3580
Cell Death Detection ELISA	Roche	Cat. No. 11 774 425 001
Ethidium homodimer-1)	Thermo Fisher Scientific	E1169
Calcein	Thermo Fisher Scientific	C3100MP
Software and Algorithms		
Confocal microscopy software	ZEN2012	https://www.zeiss.com/microscopy/int/products/microscope-software/zen-lite.html
GraphPad Prism 7.0	GraphPad Software	https://www.graphpad.com/scientific-software/prism

CONTACT FOR REAGENT AND RESOURCE SHARING

Further information and requests for resources and reagents should be directed to the Lead Contact, Albert Salehi (s_albert.salehi@med.lu.se).

EXPERIMENTAL MODEL AND SUBJECT DETAILS

Cell line

INS-1 832/13 cells derived from male rat insulinoma were kindly donated by Dr. C. B. Newgaard, Duke University, (USA).

Human Islet Preparations

Human pancreatic islets were obtained through collaboration between Human Tissue Laboratory within Lund University Diabetes Centre (LUDC) and the Nordic Network for Clinical Islet Transplantation (Prof. Olle Korsgren, Uppsala University, Sweden). Donors were grouped according to HbA1c i.e. less than 6% (ND), between 6 % and 6.5 % (IGT), higher than 6.5% or history of diabetes (T2D) (See Table S1). The human islets (70-90 % purity) had been cultured in CMRL 1066 (ICN Biomedicals, Costa Mesa, CA) supplemented with 10 mM HEPES, 2 mM L-glutamine, 50 μg/ml gentamicin, 0.25 μg/ml fungizone (Gibco, BRL, Gaithersburg, MD), 20 μg/ml ciprofloxacin (Bayer Healthcare, Leverkusen, Germany) and 10 mM nicotinamide at 37°C (5% CO2) for 1 to 5 days prior to the arrival in the laboratory. The islets were then hand-picked under stereomicroscope prior to use. All procedures using human islets were approved by the ethical committees at Uppsala and Lund Universities, Sweden.

Mouse Islet Preparations

5 weeks old Female db/db (on the B6/KS genetic background) mice and control (C57/bl) weighing 18-25 g, were from Janvier Laboratory, France). The experimental procedures were approved by the Ethics Committee for Animal Research at Lund University. The animals were given a standard pellet diet (B&K) and tap water ad libitum. Isolation of pancreatic islets was performed by retrograde injection of a collagenase solution via the pancreatic duct and islets were then collected under a stereomicroscope at room temperature.

METHOD DETAILS

Cell Culture

INS-1 832/13 cells were cultured in RPMI-1640 containing 11.1 mM D-glucose and supplemented with 10% fetal bovine serum, 100 U/ml penicillin (Gibco), 100 μg/ml streptomycin (Gibco), 10 mM N-2 hydroxyethylpiperazine-N'-2-ethanesulfonic acid (HEPES),

2 mM glutamine, 1 mM sodium pyruvate, and 50 μM β-mercaptoethanol (Sigma), at 37l C in a humidified atmosphere containing 95% air and 5% CO_2. For VDAC1 over-expression by VDAC1 plasmid (1 μg/ml) and VDAC2 down-regulation by and siRNA, INS1 cells were cultured to 60% confluence. To study the long-term effects (24-72h) of high glucose (20 mM) (glucotoxicity) compared to basal glucose (5 mM), INS-1 cells were cultured with RPMI 1640 complete media (Ahmed et al., 2010) in the presence or absence of indicated agents and thereafter VDAC1 expression levels, cell viability and function were measured.

Plasmid and Transient Transfections

INS1 cells were seeded in six-well plates at a density of ~5 x 10^5 cells in culture medium without antibiotics and transfected with full-length cDNA encoding prevalidated *VDAC1* construct (1 μg/ml plasmid) encoding VDAC1 or control plasmid (non-coding) using Effectene Transfection Reagent (Qiagen) according to the manufacturer's instructions. Tween four post transfection, medium was replaced with fresh medium containing antibiotics. If not indicated otherwise, at 72h post-transfection, the cells were harvested and analyzed by immunoblotting and qPCR for the relative level of various proteins and mRNA.

Histone Acetyltransferase p300 (Ep300) Knock- out Cells

Histone acetyltransferase p300 (Ep300) was knocked out in the rat pancreatic β-cell line (INS1 832/13) by CRISPR/Cas9 as recently described (Bompada et al., 2016).

Insulin Secretion in Cultured INS-1 Cells

Cells with VDAC1 over-expressed or VDAC2 down-regulated were used to measure the insulin secretion. To this end, INS-1 cells were kept in HEPES balanced salt solution (HBSS; 114 mM NaCl; 4.7 mM KCl; 1.2 mM KH2PO4; 1.16 mM MgSO4; 20 mM HEPES; 2.5 mM CaCl2; 25.5 mM NaHCO3; 0.2% BSA, pH 7.2) supplemented with 2.8 mM glucose for 2 h at 37°C. Thereafter the cells were incubated for 1 h in the fresh medium with the denoted glucose concentrations and test agents. After incubation an aliquot of medium was removed for analysis of secreted insulin and cellular insulin content was measured as for islets (see below).

Conductance Measurement by Patch Clamp Recording

For electrophysiological experiments, INS-1 cells were transiently co-transfected with plVDAC1 and eGFP. Both intensely and less Fluorescent cells were selected for patch clamping. Whole-cell currents were evoked and recorded by EPC10 amplifier and Pulse software (HEKA, Lambrecht/Pfalz, Germany) as previously described (Buda et al., 2013) with the temperature maintained at 32°C. The cells were continuously perfused with extracellular solution containing 118 mM NaCl, 20 mM tetraethylammonium chloride, 5.6 mM KCl, 2.6 mM $CaCl_2$, 1.2 mM $MgCl_2$, 5 mM HEPES and 5 mM glucose (pH 7.4 with NaOH) in the presence of 100 uM tolbutamide (T0891, Sigma-Aldrich). The intracellular solution consisted of 125 mM Cs-glutamate, 10 mM CsCl, 10 mM NaCl, 1 mM $MgCl_2$, 5 mM HEPES, 3mM Mg-ATP, 0.1 mM cAMP and 0.05 mM EGTA (pH 7.2 with CsOH). 20 uM metformin was used for perfusion, and approximately 8 mM metformin or 130 nM VDAC1 antibody (both diluted approximately 100 fold) for acute addition as indicated in figure legends or text. Conductance was measured by applying 200-ms voltage ramps from -90mV to -50mV. In the experiments studying the effects of acute addition of VDAC1 ab or metformin (Figures S5C and S5D), conductance was measured continuously in the same single cell before, during and after the acute addition of the respective compounds (anti-VDAC1 antibodies or metformin). Data presented are from 15 s after the addition when steady-state was reached.

For experiments in Figure 5D, the cell dish in the experimental chamber was continuously exposed to 20 μM metformin. Conductance measurements were performed after at least 5 minutes exposure, and the cell dish was replaced no later than after 1 hour of perfusion. No wash-out experiments were performed in this experiment.

VDAC Current Measurement on the Reconstituted VDAC1 in Planar Lipid Bilayer (PLB)

VDAC1 was purified from rat liver mitochondria as described previously (Ben-Hail et al., 2016). To measure single and multiple channel current, a planar lipid bilayer (PLB) was prepared from soybean asolectin dissolved in n-decane (30 mg/ml). Purified VDAC1 (10–100 ng) was added to the chamber defined as the cis side containing 0.5 M NaCl. After one or a few channels were inserted into the PLB, excess protein was removed by perfusing the cis chamber with ~10 volumes of solution to prevent further channel incorporation. Following several recordings of channel activity at different voltages, metformin or VBIT-4 was added to the cis chamber, and currents through the channel were again recorded. Currents were recorded by voltage-clamping using a Bilayer Clamp BC-535B amplifier (Warner Instruments, Hamden, CT). Currents were measured with respect to the *trans* side of the membrane (ground). The currents were low pass-filtered at 1 kHz and digitized online using a Digidata 1440-interface board and Clampex software (Axon Instruments, Union City, CA). Analysis was done using pClamp 10.2 software (Axon Instruments, Union City, CA), or excel (Microsoft).

VDAC1 Binding Study

The binding study on the purified VDAC1 channel protein performed using a Nano-Temper Monolith NT.115 apparatus as described previously (Ben-Hail et al., 2016). Briefly, purified VDAC1 was fluorescently labeled using NanoTemper Protein labeling kit BLUE (L001, NanoTemper Technologies). A constant concentration of the protein was incubated with different concentrations of the tested inhibitor in PBS. Afterward, 3–5 μl of the samples were loaded into a glass capillary (Monolith NT Capillaries), and thermophoresis

analysis was performed (LED 20%, IR laser 20%). The results are presented as % of the bound fraction calculated as follows: fraction bound100x(F - F min)/(F max-F min).

Intraperitoneal Glucose Tolerance Tests (IPGTT)

IPGTTs were performed in db/db and c57/bl mice after treatment with VDAC1 blocker (daily intraperitoneal injection with VBIT-4, 25 mg/kg body weight) for 5 weeks. Prior to IPGTT test, the mice were fasted for 4 h. Glucose was dissolved in 0.9% NaCl and 2.0 g glucose/kg body weight was injected intraperitoneally (total volume load was 0.3 ml). Serial blood sampling thereafter from vena saphena was performed at 0, 5, 15, 30 and 90 min as previously described elsewhere (Bowe et al., 2014). Blood glucose was analyzed using glucose oxidase method and plasma insulin was analyzed by ELISA. The Cumulative (area under the curve) changes in plasma glucose or insulin were calculated by subtracting the recorded values from basal (time 0). The homeostasis model assessment of insulin resistance (HOMA-IR) (insulin (mU/l)*glucose (mmol/l)/22.5) was used to calculate insulin sensitivity during the IPGTT as described (Bowe et al., 2014).

Glucose-stimulated Insulin Secretion (GSIS) in Human and Mouse Islets

Human pancreatic islets were collected under a stereomicroscope at room temperature and cultured at 5 or 20 mM glucose in the absence or presence of test agents for 72 h. Thereafter the islets were washed and preincubated for 30 min at 37°C in Krebs Ringer bicarbonate buffer (KRB), pH 7.4, supplemented with HEPES (10 mM), 0.1% bovine serum albumin, and 1 mM glucose. Each incubation vial contained 12 islets in 1.0 ml KRB buffer solution and treated with 95% O_2 and 5% CO_2 to obtain constant pH and oxygenation. After preincubation, the buffer was changed to a medium containing either 1 mM or 16.7 mM glucose. The islets were then incubated for 1 h at 37°C in a metabolic shaker (30 cycles per min). Immediately after incubation an aliquot of the medium was removed for analysis of insulin, and the islets were incubated in acid-ethanol for insulin content determination by radioimmunoassay (Wiederkehr et al., 2011).

Quantitative Polymerase Chain Reaction (qPCR)

Total RNA from handpicked mouse islets, human donor islets (Diabetic and non-diabetic) or INS1 cells were extracted using RNAeasy (Qiagen, Hilden, Germany). RNA (0.5 μg) was used for cDNA synthesis with SuperScript (Invitrogen, Carlsbad, CA, USA). Concentration and purity of total RNA was measured with a NanoDrop ND-1000 spectrophotometer (A260/A280>1.9 and A260/A230>1.4) (NanoDrop Technologies, Wilmington, DE) and RNA Quality Indicator (RQI) higher than 8.0 (Experion Automated Electrophoresis, Bio-Rad, USA) was considered to be high-quality total RNA preparations. A 10 μl of reaction mixture with 20 ng cDNA, 5 μl TaqMan mastermix (Applied Biosystems, Foster City, CA, USA), and 100 nM TagMan gene expression assay were run in a 7900HT Fast Real-Time System (Applied Biosystems). The qPCR was carried out as follows: 50°C for 2 minutes, 95°C for 10 minutes, 40 cycles of 95°C for 15 seconds, and 60°C for 1 minute. The amount of mRNA was calculated relative to the amount of housekeeping genes (GAPDH, PPIA or HPRT) mRNA in the same sample by the formula X0/R0 = 2CtR-CtX, where X0 is the original amount of mRNA for the gene of interest, R0 is the original amount of HPRT mRNA, CtR the Ct value for HPRT, and CtX the Ct value for the gene of interest. qPCR results were normalized to housekeeping genes. Primer sequences used in the qPCR assays are provided in Table S2.

To measure the expression level of VDAC1 and VDAC2 under glucotoxic (20 mM glucose) compared to normal condition (5 mM glucose), human islets (300 islets/30 mm Dish) were cultured in RPMI 1640 medium containing either 5 or 20 mM glucose with or without indicated test agents in a humidified incubator (37°C, 5% CO_2) for 24 to 72 h. VDAC1 and VDAC2 mRNA and protein levels were investigated by both qPCR (relative to GAPDH, HPRT or PPIA) and Western blotting (relative to the expression of and β-actin), respectively.

Immunoblotting

Human islet or INS1 832/13 cells were suspended in 100 μl of SDS-buffer (50 mM Tris-HCl, 1mM EDTA) supplemented with complete protease inhibitor cocktail (Roche, Basel Switzerland), frozen and sonicated on ice on the day of analysis. The protein content of the homogenates was determined by commercially available kit according to the manufacturer's recommendation (Thermo Scientific, USA). Homogenate samples of islets and INS1 cells representing 30 μg of total protein were subjected to 7.5% SDS-polyacrylamide gels (Bio-Rad, Hercules, CA, USA). After electrophoresis, proteins were transferred into nitrocellulose membranes (Bio-Rad, Hercules, CA, USA). The membranes were blocked in LS-buffer (10 mM Tris, pH 7.4, 100 mM NaCl, 0.1% Tween-20) containing 5% non-fat dry milk powder for 40 min at 37°C. Subsequently the membranes were incubated over night with rabbit-polyclonal anti-VDAC1 and goat-polyclonal anti-VDAC2 antibodies (1:500) at room temperature. After washing (three times) in LS-buffer the membranes were incubated with horseradish peroxidase-conjugated anti-goat or anti-rabbit antibodies (1:1000). Immunoreactivity was detected using an enhanced chemiluminescence reaction (Pierce, Rockford, IL, USA). The band-intensity was related to β-actin.

Immunostaining and Confocal Imaging

Isolated human or mouse islets as well as INS-1 cells were seeded on glass-bottom dishes cultured overnight. Cells were then washed twice and fixed with 3% paraformaldehyde for 10 min, followed by permeabilization with 0.1% Triton-X 100 for 15 min. The blocking solution contained 5% normal donkey serum in PBS and was used for 15 min. Primary antibodies against mouse VDAC1 (Abcam), goat VDAC2 (Abcam) and Guinea pig insulin (Eurodiagnostica) were diluted in blocking buffer and incubated

overnight at 4°C. Immunoreactivity was quantified using fluorescently labeled secondary antibodies (1:200) and visualized by confocal microscopy (Carl Zeiss, Germany). The ratio is calculated by mean intensity of plasma membrane to mean intensity in cytosol, according to the formula: $\frac{\left[\frac{(i1*a1)-(i2*a2)}{a1-a2}\right]}{\left[\frac{(i2*a2)-(i3*a3)}{a2-a3}\right]}$. Where i1, i2 and i3 represent the intensities of whole cell, cytosol and nucleus, a1, a2 and a3 represent the area of whole cell, cytosol and nucleus respectively (Buda et al., 2013).

Immunohistochemistry was performed following standard protocol. Briefly, the human pancreases were paraffin embedded, cut into 10 um thick sections and rehydrated for staining with primary antibodies of guinea pig anti-insulin (Eurodiagnostica), rabbit monoclonal anti-VDAC1 (Abcam) and mouse monoclonal anti-SNAP25 (SYSY) were used to detect insulin, VDAC1 and SNAP-25 expression, respectively. The nuclei were stained by Hoechst 33258. The human pancreatic sections from ND and T2D donors were kindly provided by the Nordic Network for Islet transplantation (Prof. Olle Korsgren, Uppsala, Sweden). The images were acquired by confocal microscopy and the colocalization analysis of VDAC1 and SNAP25 in insulin-staining cells was performed with software ZEN2012.

Single Cell ATP/ADP Ratio Measurement

For single cell ATP/ADP ratio measurements, INS-1 cells were co-transfected as above with either wtVDAC1 or des-Cys(127/232) VDAC1 plasmid (Aram et al., 2010) together with PercevalHR (1 μg/ml) each. Single cell imaging was performed by confocal microscopy as described (Tantama and Yellen, 2014) and applied to INS-1 cells. Expression levels were determined by qPCR.

TIRF Microscopy

INS-1 cells were seeded on glass-bottom dishes and transfected with wild-type VDAC1-EGFP and des-Cys(127/232)VDAC1-EGFP for 48 hours. The membrane expression of wild type and des-Cys(127/232)VDAC1 was measured by TIRF imaging, which detects the VDAC1 signal about 150 nm close to the glass surface. The analysis of VDAC1 spots was performed by ImageJ Plugin and ZEN2012 software. The experiments were repeated 3 times with 30 cells in each group of wild type and des-Cys(127/232)VDAC1transfected cells.

Small Interfering RNA (siRNA) for Protein Expression Silencing

For VDAC1 and VDAC2 small interfering RNA (siRNA) experiments, 20-25 nucleotide stealth prevalidated siRNA duplex designed for rat Vdac1 and Vdac2 (Applied Biosystem) were used. INS1 cells were seeded in six-well plates at a density of $\sim 5 \times 10^5$ cells in culture media without antibiotics and transfected with DharmaFECT1 (Dharmacon; Lafayette, CO, USA) according to the manufacturer's instructions. Cells were transfected for 24 h with the Vdac1 and Vdac2 siRNA at a final concentration of 50 nM or with control siRNA (non-targeting siRNA) at the same concentration before changing to fresh media including antibiotics. At 72 h after transfection, cells were lysed to extract total RNA or protein to measure the knockdown efficacy.

For silencing Chrebp and Txnip expression in INS1 832/13 cells, 20-25 nucleotide stealth prevalidated siRNA duplex designed for rat Chrebp and Txnip (Applied Biosystem) were used, following the same protocol as for VDAC silencing. At 72h after transfection, cells were lysed to extract total RNA or protein to measure Chrebp and Txnip knockdown efficiency as well as VDAC1 expression.

Silencing by shRNA Mediated (Lentivirus) in Human Islets

Specific silencing of endogenous human hVDAC1 or hVDAC2 was achieved using lentiviral based shRNA-silencing technique (Santa Cruz, CA, USA). Isolated human islets were incubated at 2.8 mM glucose plus Polybree for 90 min. Thereafter the medium was removed and the islets were washed before addition of culture medium with lentiviral particle containing VDAC1-shRNA or VDAC2-shRNA (5 μl/ml) and the islets were cultured for 72 h at 5 or 20 mM glucose. For comparison, scramble (lentiviral particles without targeting any specific region) served as control. After the culture period the medium was removed and the islets were dispersed into single cells and subjected to cell viability assay using MTS reagent kit.

Measurement of Cellular Reductive Capacity (MTS), Apoptosis and Viability

The reductive capacity of cells was measured either on INS-1 cells or dispersed human islet cells when the INS-1 cells or islets were subjected to 5 or 20 mM glucose for 72 h in the absence or presence of test agents or after down-regulation of VDAC1 and VDAC2 as described elsewhere. Measurement of reductive capacity was performed using the MTS reagent kit according to the manufacturer's instructions (Promega) and (Janjic and Wollheim, 1992). Apoptosis was measured with the Cell Death Kit (Roche Diagnostics), which quantifies the appearance of cytosolic nucleosomes.

To measure the cell viability in living cells, we used EthD1 (Ethidium homodimer-1) and calcein to indicate death and live cell in INS-1 cells, respectively according to manufacturer (Thermo Fisher, USA). The plasma membrane targeted VDAC1 (plVDAC1) and mitochondrial VDAC1 (mtVDAC1) were overexpressed in INS-1 cells cultured with either 5 mM glucose (5G) or 20 mM glucose (20G). The mean intensity of Ethidium and Calcein were calculated to indicate live and death cells, respectively.

Measurement of Oxygen Consumption Rate (OCR)

OCR was measured in INS-1 832/13 cells using the XF (extracellular flux) analyzer XF24 (Seahorse Bioscience), as previously described in detail (Wiederkehr et al., 2011). An assay medium composed of 114 mMNaCl, 4.7 mM KCl, 1.2 mM KH_2PO4,

1.16 mM $MgSO_4$, 20 HEPES, 2.5 $CaCl_2$, 0.2% bovine serum albumin, pH 7.2, and supplemented with 2.8 mM glucose was used in the XF analysis. The cells were seeded in an XF24 24-well cell culture microplate at 2.5×10^5 cells/well (0.32 cm^2 growth area) in 500 µl of growth medium and incubated overnight at 37°C in a humidified atmosphere of 95% air and 5% CO_2. Prior to assay, RPMI 1640 medium was removed and replaced with 750 µl of assay medium. The cells were preincubated under these conditions for 2 h at 37°C in air. The experiments were designed to determine respiration in low (2.8 mM) glucose and for 60 min following the transition to high (16.7 mM) glucose. The proportions of respiration driving ATP synthesis and proton leak were determined by the addition of oligomycin (4 µg/ml). After a further 30 min, 4 µM of dinitrophenol was added to determine maximal respiratory capacity. After a further 10 min, 1 µM rotenone was added to block transfer of electrons from complex I to ubiquinone.

ATP Determination

ATP content and release from isolated mouse or human islets or from INS-1 cells after transfection with mitochondrial targeted VDAC1 (mtVDAC1) or plasma membrane targeted VDAC1 (plVDAC1) plasmids was determined using a luminometric assay kit according to manufacturer's recommendation (Biovision). After incubation of islets (50/vial) or INS-1 cells for 60 min, an aliquot of the media was removed for subsequent measurements of released ATP. Then the islets or INS-1 cells were washed 3 times and the lysates were used for measurements of ATP and protein contents following the protocol provided by the vendor.

Mitochondrial and Cytosolic Ca^{2+} Imaging in Single Cells

INS-1 cells (6×10^5 cells) were seeded on 24-well plates and subjected to the following treatments: Control with 5 mM glucose, VDAC1 overexpressed (OE) and silencing VDAC2 (KD) by siRNA in 5 mM glucose culture, and 3 days treated with 20 mM glucose. 24 hours prior to Ca^{2+} imaging, the cells were transferred to glass-bottom dishes while diluted 1:6 (~1×10^5 cells). The cells were loaded 1 h with Rhod-2 (0.75 uM) and Fluo-5F (0.5 uM) dissolved in the perfusion buffer (KRB). Time lapse ROi images were acquired by confocal microscopy and the mean intensity of ROIs was analyzed by ZEN 2009 software. The data calculation was performed with Excel and normalized ratio was calculated by Fi/F0 (Buda et al., 2013).

QUANTIFICATION AND STATISTICAL ANALYSIS

The results are expressed as means ± SEM for the indicated number of individual experiments (as given in figure legends) or illustrated by an observation representative of a result obtained from different experiments (Western blots). The significance of random differences were analyzed by Student's t-test or where indicated the analysis of variance followed by Tukey-Kramers' multiple comparisons test. P value <0.05 was considered significant. All data were assessed to ensure normal distribution and equal variance between different groups.

DATA AND SOFTWARE AVAILABILITY

The accession number for the deposited data is GEO: GSE108072.

Cell Metabolism, Volume *29*

Supplemental Information

Preserving Insulin Secretion in Diabetes by Inhibiting VDAC1 Overexpression and Surface Translocation in β Cells

Enming Zhang, Israa Mohammed Al-Amily, Sarheed Mohammed, Cheng Luan, Olof Asplund, Meftun Ahmed, Yingying Ye, Danya Ben-Hail, Arvind Soni, Neelanjan Vishnu, Pradeep Bompada, Yang De Marinis, Leif Groop, Varda Shoshan-Barmatz, Erik Renström, Claes B. Wollheim, and Albert Salehi

Supplemental Information

Preserving insulin secretion in diabetes by inhibiting VDAC1 overexpression and surface translocation in β-cells

Enming Zhang[1], Israa Mohammed Al-Amily[1], Sarheed Mohammed[1], Cheng Luan[1], Olof Asplund[1], Meftun Ahmed[2], Yingying Ye[1], Danya Ben-Hail[3], Arvind Soni[1], Neelanjan Vishnu[1], Pradeep Bompada[1], Yang De Marinis[1], Leif Groop[1,4], Varda Shoshan-Barmatz[3], Erik Renström[1], Claes B. Wollheim[1,4,6]* and Albert Salehi [1,6,7]**

[1]Department of Clinical Science, Malmö, University of Lund, Malmö, Sweden [2]Present address, Acandemic hospital Uppsala University, Uppsala, Sweden [3]Department of Life Sciences and the National Institute for Biotechnology in the Negev, Ben-Gurion University of the Negev, Beer-Sheva 84105, Israel, [4]Finnish Institute for Molecular Medicine, Helsinki University, Helsinki, Finland, [5]Department of Cell Physiology and Metabolism, University Medical Centre, 1 rue Michel-Servet, Geneva 4, Switzerland

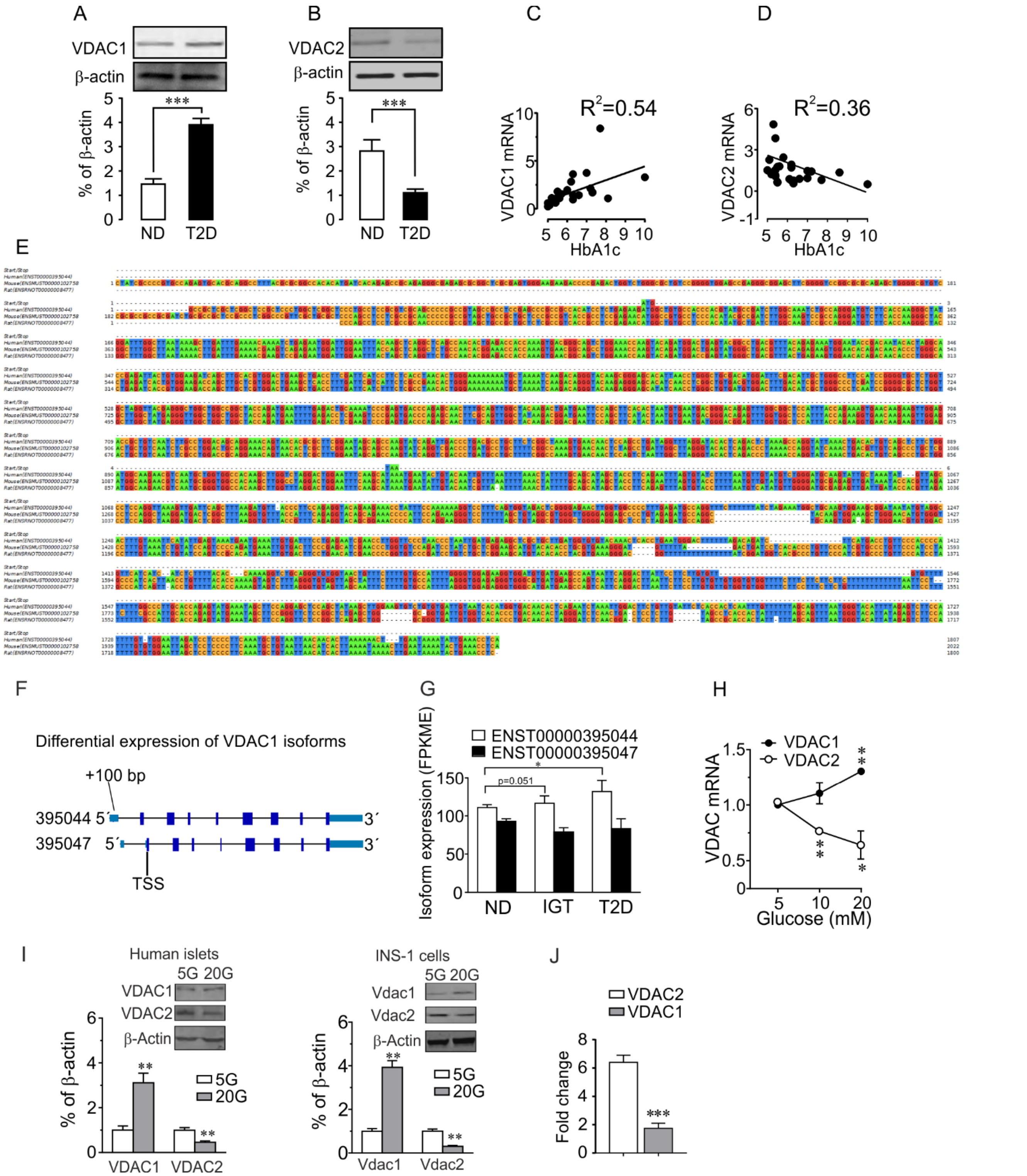

Figure S1 (related to Figure 1). A, B, Representative Western blots and fold change by densitometry normalized to β-actin (mean ± SEM) for VDAC1 (A) and VDAC2 (B) in islet extracts from non-diabetic (ND) and T2D donors (n = 8 donors in each group). C, Correlation between islet *VDAC1* mRNA and donor HbA1c in ND+T2D excluding the four metformin-treated (n=26), (*cf.* Figure 1B insert), P<0.005. D, Correlation between islet *VDAC2* mRNA and donor HbA1c in ND+T2D excluding the four metformin-treated (n=26) (*cf.* Figure 1D insert), *P<0.05. E, Alignment of the human, mouse and rat VDAC1genes. Note the difference in exon 1 in mouse on the one hand and human and the rat on the other. Alignments to the human *VDAC1* gene on chromosome 5 were visualized using the Integrative Genomics Viewer. F, Scheme of the two most abundant *VDAC1* transcript variants (ENST00000395044 and ENST00000395047) in human islets analyzed by RNA sequencing. G, Increased expression of *VDAC1* transcript variant ENST00000395047 in ND islets and those of donors with impaired glucose tolerance (IGT) and T2D as analyzed by RNA sequencing (ND=181 donors, IGT=19, T2D=16 donors). H, *VDAC1* and *VDAC2* mRNA expression in human islets cultured at 5, 10 or 20 mM glucose for 72h. Mean±SEM from two different donors with three replicates each are shown. I, Comparison of VDAC1 and VDAC2 protein levels inhuman islets and INS-1 cells after culture at 5 or 20 mM glucose for 72h. Top, representative Western blots bottom quantification of results from four different donors and from four different INS-1 cells experiments. Mean+SEM of four separate experiments normalized to the values in 5 mM glucose for islets and INS-1 cells, respectively. J, Vdac1 and Vdac2 protein detection in INS-1 cells by a rabbit polyclonal antibody, targeting a common epitope of the proteins. Mean±SEM of four separate observation. *P<0.01, ***P<0.0001.

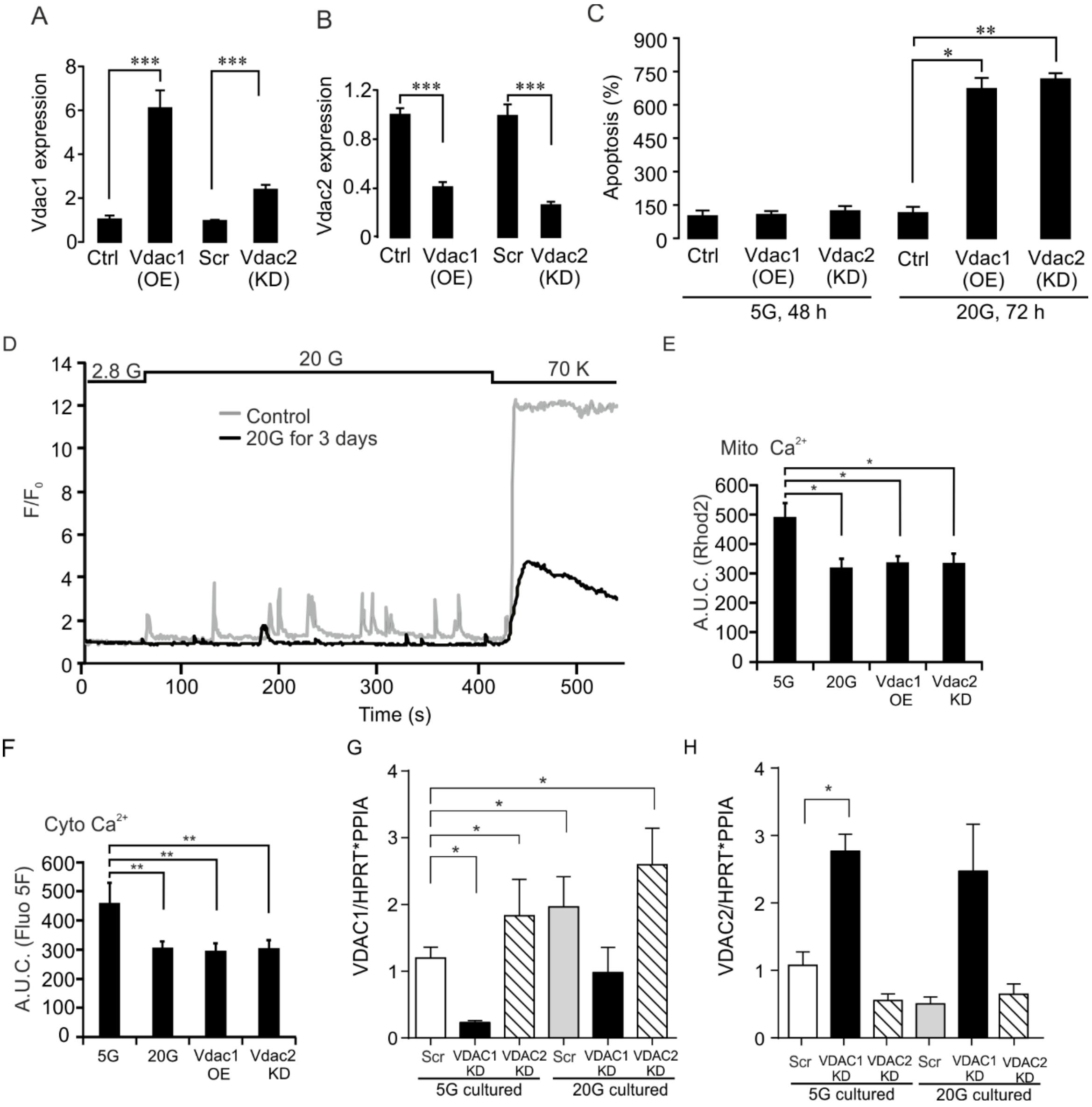

Figure S2 (related to Figure 2). A,B, Reciprocal changes in *Vdac1* (A) and *Vdac2* transcript (B) expression after overexpression of *Vdac1* (OE) and knock-down of *Vdac2* (KD) in INS-1 cells. Data are expressed as mean ± SEM (n = 9 different experiments). C, *Vdac1* overexpression (OE) or *Vdac2* knock-down (KD)-induced apoptosis measured in INS-1 cells either directly after 48 h of culture in 5 mM glucose or after an additional 72h culture in 20 mM glucose. Apoptosis is expressed as % of the control values measured in cells cultured at 5 mM glucose. Results are from three independent experiments performed with 2-3 technical replicates. D, mitochondrial (Mito) and cytosolic (Cyto) Ca^{2+} in INS-1 cells cultured at 5 or 20 mM glucose as well as after overexpression (OE) of *Vdac1* or knock-down (KD) of *Vdac2*. The cells were cultured 3 days at either 5 or 20 mM glucose. VDAC1 (OE) and VDAC2 (KD) were performed in cells cultured at 5 mM glucose. At the end of the culture period, the cells were loaded with Rhod-2 (0.75 uM) and Fluo-5F (0.5 uM) for Mito Ca^{2+} and Cyto Ca^{2+} respectively, as indicated in STAR Methods. The basal glucose concentration (2.8 mM) was raised to 20 mM glucose followed by addition of 70 mM K^+ as indicated in the figure. Representative traces of mito Ca^{2+} under control and glucotoxic conditions with indicated additions (20 mM glucose and 70 mM K^+). E, Mito Ca^{2+} as area under the curve (AUC) after acute stimulation with 20 mM glucose. F, Cyto Ca^{2+} expressed as AUC after acute stimulation with 20 mM glucose. Data are mean ± SEM of three separate experiments. Mito and Cyto Ca^{2+} were monitored simultaneously in respectively 30 cells (5 mM glucose culture), 28 cells (20 mM) culture, 27 cells with *Vdac1* OE and 29 after *Vdac2* KD. G, *VDAC1* mRNA levels in human islets after *VDAC1*-KD or *VDAC2*-KD. H, Same as in G for *VDAC2* mRNA levels. Data are mean ± SEM from three donors with 3 technical replicates in each. *P<0.05, **P<0.01, ***P<0.001.

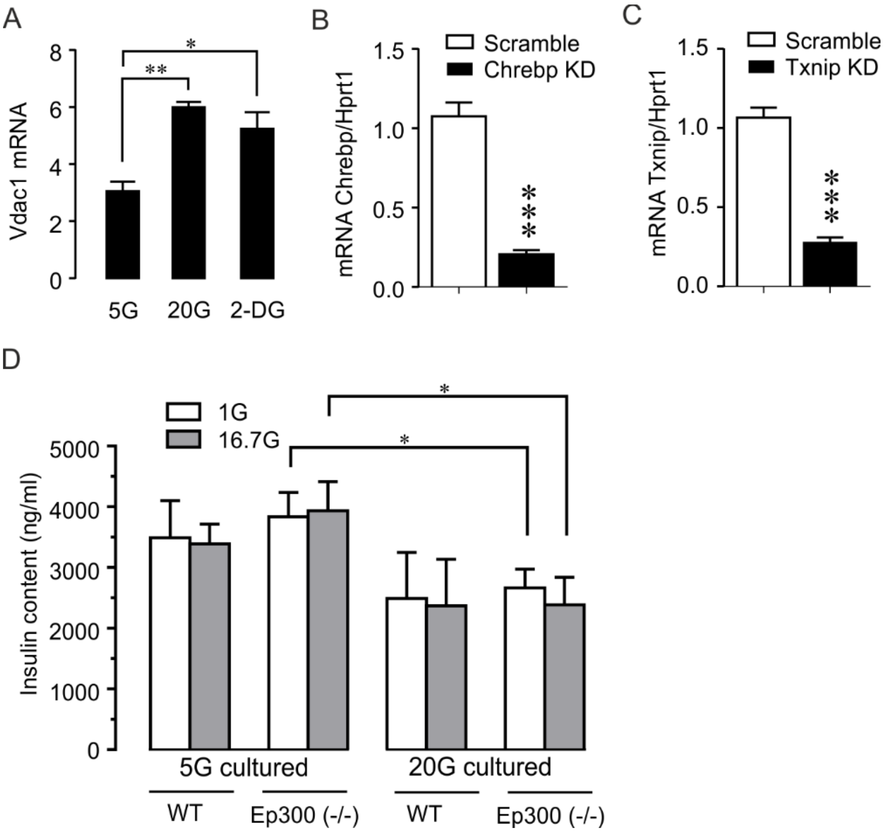

Figure S3 (related to Figure 3). A, 2-deoxy-glucose (2-DG) (15 mM) mimics the effects of glucotoxicity on *Vdac1* mRNA expression in INS-1 cells cultured at 5 mM glucose for 72h. Data are mean ± SEM from three independent experiments. B, Chrebp mRNA after knock-down in INS-1 cells (n=4 different experiments). C, Txnip mRNA after knock-down in INS-1 cells (n=4 different experiments). D, insulin content in INS-1 (Ep300(-/-) and control cells for the experiments in Figure 3G. (n= 3-4). *P<0.05, **P<0.01, ***P<0.001.

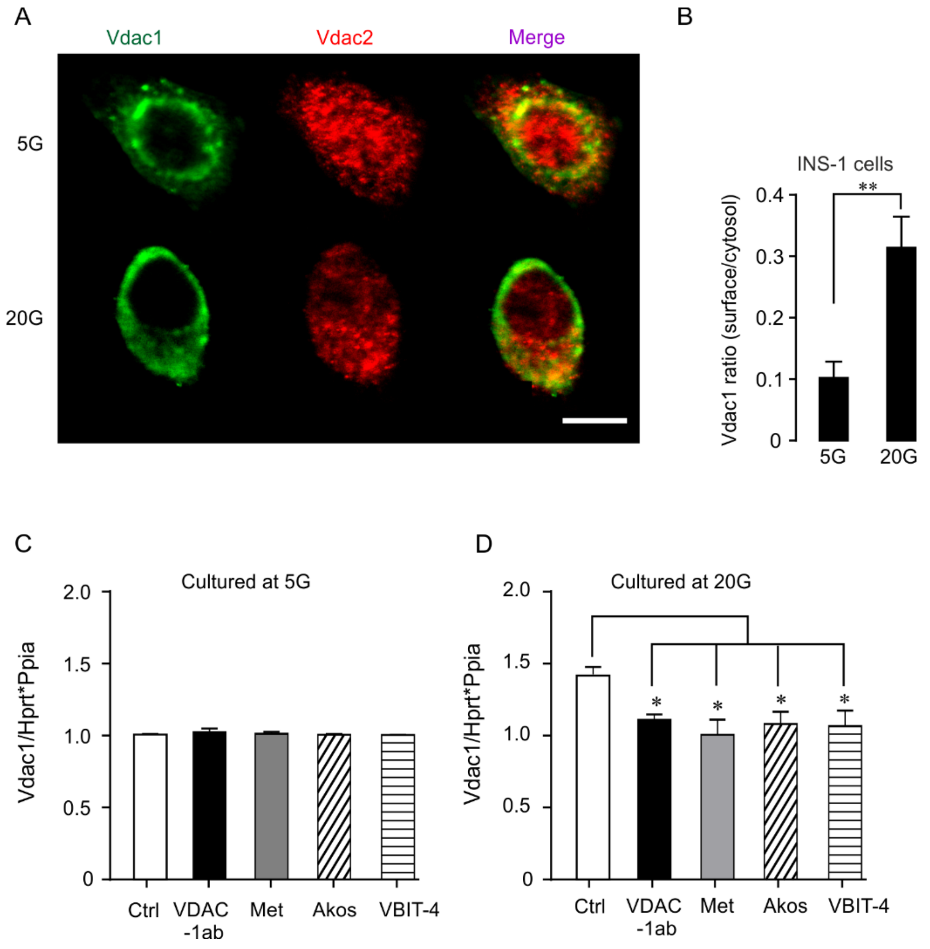

Figure S4 (related to Figure 4). A, Representative confocal images of Vdac1 or Vdac2 in INS-1 cells cultured at 5 (5G) or 20 mM glucose (20G) for 72h. The cells were double immunostained for Vdac1 and Vdac2. B, Cell surface expression of Vdac1 is calculated as the ratio of Vdac1 within 500 nm from the cell membrane over cytosolic Vdac1 (n= 7 replicates in each group from three independent experiments). C, D, VDAC1 inhibitors prevent glucotoxicity-evoked Vdac1 induction. Vdac1 expression in the presence or absence of VDAC1 antibody (VDAC1ab, 10 nM), metformin (20 µM) (Met), AKOS022075291 (20 µM) (AKOS) or VBIT-4 (20 µM) in INS-1 cells cultured for 72 h at either 5 mM (C) or 20 mM glucose (D). Results are mean ± SEM of 3 independent experiments with four technical replicates. *P<0.01, **P<0.001.

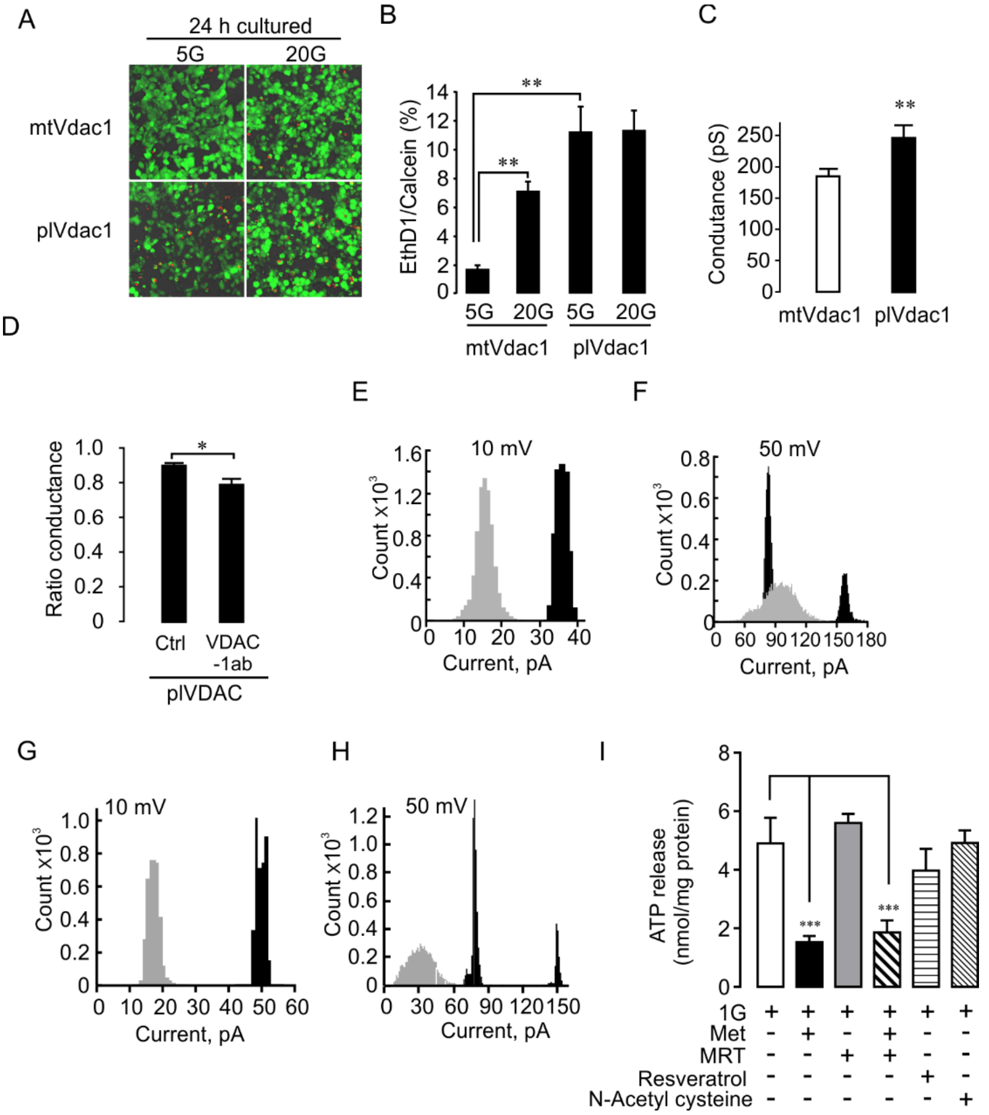

Figure S5 (related to Figure 5). A, Overexpression of plasma membrane *Vdac1* (*plVdac1*) causes cell death in INS-1 cells. Representative confocal images acquired from INS-1 cells transfected with mitochondrial *Vdac1* (*mtVdac1*) or *plVdac1* and cultured with either 5 mM (5G) or 20 mM glucose (20G) for 24h. Green (Calcein) and red (Ethidium homodimer-1, EthD1) indicate live and dead cells respectively. B, Average of ratios calculated by division of EthD1 intensity to calcein intensity under the same conditions as in A. Data are mean±SEM from three independent experiments. C, Membrane conductance recorded by the whole cell patch-clamp technique in INS-1 cells overexpressing *mtVdac1* or *plVDAC1*. Mean ± SEM of 10 cells each. D, Ratio of membrane conductance after and before acute additions of extracellular solution in INS-1 cells overexpressing *plVdac1*, without and with either VDAC1-ab or metformin. Mean ± SEM of 11 cells in each group are shown. E,F,G,H, VDAC1 conductance in planar lipid bilayer experiments. Current amplitude histograms of the experiments in Fig 5E at +10 mV (E) or 50 mV (F), before (dark grey) and 10 min after the addition of metformin (light grey) are shown. Current amplitude histograms of the experiments with VBIT-4 in Fig 5F at 10 mV (G) or, 50 mV (H), before (dark grey) and 10 min after the addition of 40 µM VBIT-4 (light grey) are shown. I, The metformin-inhibited (Met) ATP release in plVDAC1 transfected INS-1 cells is neither attenuated by an AMPK inhibitor (MRT199665, 5 µM) (MRT) (Clark et al., 2012) nor mimicked by the antioxidants resveratrol (20 µM) or N-Acetyl cystein (N-NAC 100 µM). Data are mean ± SEM of 4 independent experiments. **P<0.01.

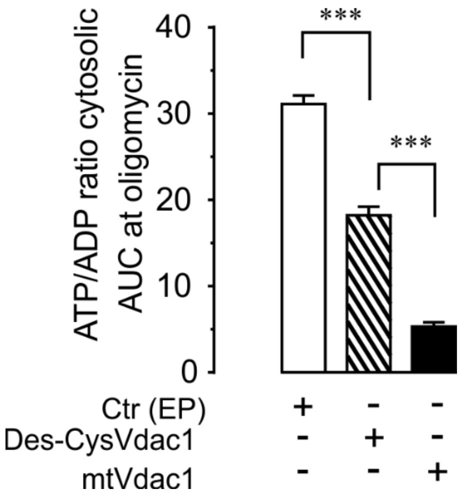

Figure S6 (related to Figure 6). The Area under curve (AUC) of mitochondrial ATP production in INS-1 cells blocked by oligomicyn (0.6 mg/ml) for the experiments shown in fig. 6E where cells express empty plasmid (EP), mtVdac1 or Des-CysVdac1. Data are mean ± SEM of 6 independent experiments with 5-10 cells in each.

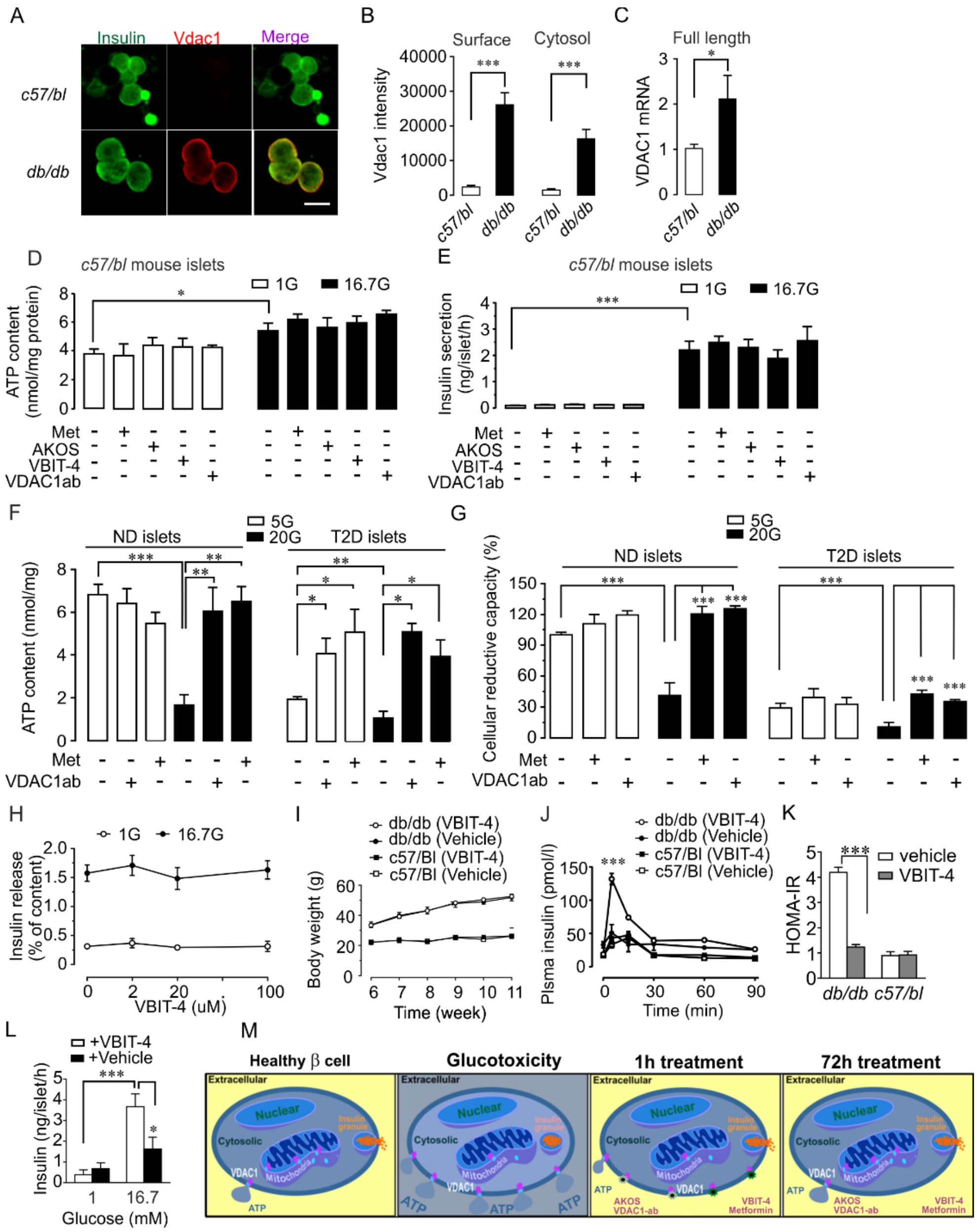

Figure S7 (related to Figure 7). Aberrant localization of VDAC1 and effects of its blockade in *db/db* mouse islets. A, Representative confocal images showed that Vdac1 is expressed predominantly on the surface of β-cells in *db/db* mouse islets (scale bar: 10 um). B, Surface and cytosolic mean intensity of Vdac1 in β-cells were measured. Results from 23 (*c57/bl*) and 25 (*db/db*) islets isolated from 8 mice of each genotype are shown. Data are mean ± SEM. C, *Vdac1* mRNA measured by qPCR in *c57/bl* and *db/db* mouse islets. D,E, VDAC1 antibody (10 nM), metformin, AKOS022075291 and VBIT-4 (all at 20 μM) do not affect ATP content (D) or GSIS (E, in *c57/bl*) islets. Islets isolated from four *c57/bl* mice were incubated separately in a single experiment. Data are mean ± SEM. F, ATP content in isolated islets from non-diabetic (ND) and T2D organ donors after culture for 72h at 5 (5G) or 20 mM glucose (20G) in the presence or absence of VDAC1 antibody (10 nM) (VDAC1ab) or metformin (Met, 20 μM). Note the improved ATP content in glucotoxic condition and T2D islets. Data are mean ± SEM of 5 non-diabetic and 3 T2D donors. G, Cellular reductive capacity (formazone production) measured in non-diabetic or T2D islets (F) after 5 or 20 mM glucose culture as in F. Mean ± SEM of 5 non-diabetic and 3 T2D donors are shown. H, Effect on insulin secretion of different concentrations of VBIT-4 added during 1 h incubation in the presence of 1 or 16.7 mM glucose (16.7G) in ND human islets. Mean+SEM of two experiments with 1 donor each. I, Body weight of *db/db* and *c57/bl* mice treated with VBIT-4 (25 mg/kg ip) or vehicle for 5 weeks. J, Plasma insulin during IPGTT (2 g/kg) of the experiments shown in Fig. 7H. Mean±SEM 12 *db/db* in each group and 5-6 c57/bl in each group is shown. K, Insulin sensitivity in *db/db* mice is improved by VBIT-4. HOMA-IR was calculated from the results shown in figures 7H and S7J. Mean+SEM of 12 *db/db* (each group) and 5-6 *c57/bl* mice are shown. L, Glucose-stimulated insulin secretion from isolated islets of *db/db* mice treated for 5 weeks with daily IP injections of VBIT-4 (25 mg/kg) or vehicle. The islets were incubated for 1h at 1 (1G) or 16.7 mM glucose (16.7G). Data are mean ± SEM of 6 mice in each group with 2 technical replicates. * p<0.05, ** p<0.01, *** p<0.001. M, Schematic illustration of VDAC1 plasma membrane expression in β-cells of T2D islets causing cellular ATP depletion with consequent blunting of GSIS. Inhibitors of VDAC1 (VDAC1 Antibody, VBIT-4, AKOS and metformin) block ATP depletion when added acutely and restore GSIS. Moreover, 72h presence of these inhibitors prevents the VDAC1 upregulation and mistargeting to the β-cell plasma membrane under glucotoxic conditions. This effect also provides an explanation for the protracted increase in blood glucose of *db/db* mice after cessation of VBIT-4 treatment (*cf*. Figure 7G).

Table S1 (related to Figure 1): Donors grouping information based on the HbA1c and history of T2D. Mean±sem is shown.

	Number	Gender	Age (year)	BMI (index)	Hb1Ac (%)
ND	10	Male	55.7±1.5	23.7±0.2	5.5±0.04
	5	Female	58±1.2	23.2±0.34	5.6±0.06
IGT	1	Male	68	29.3	6.1
T2D	10	Male	62.6±1.9	26.5±0.9	7.0±0.3
	5	Female	59.8±3.1	30.2±1.0	6.8±0.2
